# Soybean LEC2 Regulates Subsets of Genes Involved in Controlling the Biosynthesis and Catabolism of Seed Storage Substances and Seed Development

**DOI:** 10.3389/fpls.2017.01604

**Published:** 2017-09-20

**Authors:** Sehrish Manan, Muhammad Z. Ahmad, Gaoyang Zhang, Beibei Chen, Basir U. Haq, Jihong Yang, Jian Zhao

**Affiliations:** ^1^National Key Laboratory of Crop Genetic Improvement, Huazhong Agricultural University Wuhan, China; ^2^State Key Lab of Tea Plant Biology and Utilization, College of Tea and Food Science and Technology, Anhui Agricultural University Hefei, China

**Keywords:** seed storage substances, transcription factor, *LEAFY COTYLEDON2*, triacylglycerol, carbohydrate catabolism, protein biosynthesis

## Abstract

Soybean is an important oilseed crop and major dietary protein resource, yet the molecular processes and regulatory mechanisms involved in biosynthesis of seed storage substances are not fully understood. The B3 domain transcription factor (TF) LEC2 essentially regulates embryo development and seed maturation in other plants, but is not functionally characterized in soybean. Here, we characterize the function of a soybean LEC2 homolog, GmLEC2a, in regulating carbohydrate catabolism, triacylglycerol (TAG) biosynthesis, and seed development. The experimental analysis showed that GmLEC2a complemented Arabidopsis *atlec2* mutant defects in seedling development and TAG accumulation. Over-expression of *GmLEC2a* in Arabidopsis seeds increased the TAG contents by 34% and the composition of long chain fatty acids by 4% relative to the control seeds. Transcriptome analysis showed that ectopic expression of *GmLEC2a* in soybean hairy roots up-regulated several sets of downstream TF genes *GmLEC1, GmFUS3*, Gm*ABI3, GmDof11 and GmWRI1* that regulate the seed development and production of seed storage substances. *GmLEC2a* regulated the lipid transporter genes and oil body protein gene *OLEOSIN (OLE1)*. The genes involved in carbohydrate biosynthesis and storage, such as sucrose synthesi*s*, and catabolism of TAG, such as lipases in *GmLEC2a* hairy roots were down-regulated. GmLEC2a targeted metabolic genes for seed protein in soybean.

## Introduction

Soybean is an economically important oilseed crop that is grown worldwide for the production of high quality oils and proteins in their seeds. It is unique among legumes for its 40% protein and 20% oil content on dry weight basis (Hajduch et al., 2005; Clemente and Cahoon, 2009). Besides, 70% of the soybean meal is utilized as a fodder for live stocks due to its high protein content (Chaudhary et al., 2015). Moreover, it is a potential source for pharmaceutical and fuel industry (Haslam et al., 2016). The seed storage reserves such as protein, triacylglycerol, (TAG), and starch, are filled during the seed development, which critically determine seed quality traits of many crops. Understanding of the seed development and storage substance filling into the seed thus is essential for enhancement of crop yield and nutrition quality (Fatihi et al., 2016). Efforts have been devoted to study these aspects in model plants and important crops, such as transcriptional regulation of seed development and seed filling and roles of hormones during the processes (Rosche et al., 2005; Manan et al., 2016). Transcription factors (TFs) such as Leafy cotyledon 1 (LEC1), Leafy cotyledon 2 (LEC2), Abscisic acid insensitive 3 (ABI3), FUSCA 3 (FUS3), and Wrinkled 1 (WRI1), as well as other activators or repressors of seed development or storage substance filling, have been studied, although more details and mechanisms yet to be determined, particularly when applied to important crops, such as soybean (Manan et al., 2016; Zhang et al., 2017). The molecular basis for the often observed correlations between protein, oil, and carbohydrate biosynthesis and accumulation in soybean seed is yet to be completely explored (Bates and Browse, 2012; Zhang et al., 2017), which has been one of the obstacles in soybean yield and nutrition improvement.

LEC2, a B3 DNA binding domain TF, is known to have a central regulatory role in embryo development and seed maturation in Arabidopsis, maize and castor bean (Braybrook and Harada, 2008; Kim et al., 2014; Grimault et al., 2015). LEC2 regulates other TFs, such as LEC1 and FUS3, which contribute to the development of a regulatory network of cotyledon prototype (Meinke, 1994). Arabidopsis LEC2 (AtLEC2) positively regulates seed storage protein and oil biosynthesis genes when expressed in the vegetative organs (Stone et al., 2008). The *atlec2* loss of function mutant mature seeds profiling shows a 30% reduction in oil and 15% less protein, while maintaining higher levels of sucrose and starch than the wild-type plant (Angeles-Núñez and Tiessen, 2011). LEC2 is known to have an important role in altering the relative fatty acid (FA) composition and TAG accumulation in plant tissues besides its various regulatory functions during embryogenesis, metabolic pathways, and development. The potential of LEC2 was evaluated by regulating the networks in vegetative tissues that are usually only present in seeds. AtLEC2 inducible expression increased the total FA accumulation in tobacco leaves by 6.8% (Andrianov et al., 2010). The *AtLEC2* gene induced TAGs accumulation and changed the FA composition in vegetative tissues of Arabidopsis by up-regulating *LEC1, ABI3, FUS3*, and *WRI1* gene expression (Kim et al., 2015). As a master regulator, LEC2 also provokes somatic embryo formation, and thus mutually interact with auxin and ethylene response factors (Wójcikowska et al., 2013; Nowak et al., 2015).

Here, we have characterized one of the two soybeans LEC2 (GmLEC2) homologs, GmLEC2a, from soybean genome that shows significant identity to Arabidopsis LEC2. As a soybean ortholog of AtLEC2, GmLEC2a complemented Arabidopsis *lec2* mutant phenotypes in seedling development and TAG accumulation. Over-expression of *GmLEC2a* in Arabidopsis increased the seed TAG contents. The ectopic expression of *GmLEC2a* in soybean hairy roots enhanced the TAG biosynthesis. Transcriptome analysis of GmLEC2a hairy roots in comparison to control showed that GmLEC2a up-regulated the expression of TFs, FA, and TAG metabolic genes. Interestingly, GmLEC2a over-expression also negatively regulated several phospholipid and non-polar lipid transporter genes such as FAX1 and TGDs, as well as TAG lipases. GmLEC2a specifically regulated the seed storage protein and starch biosynthesis genes in soybean hairy roots. These results provide new insights into understanding the functions of GmLEC2 in soybean, suggesting that *GmLEC2* could be a major target for metabolic engineering to produce customized soybean to meet the special demands.

## Results

### Identification of LEAFY COTYLEDON 2 from Soybean

**FIGURE 1:**
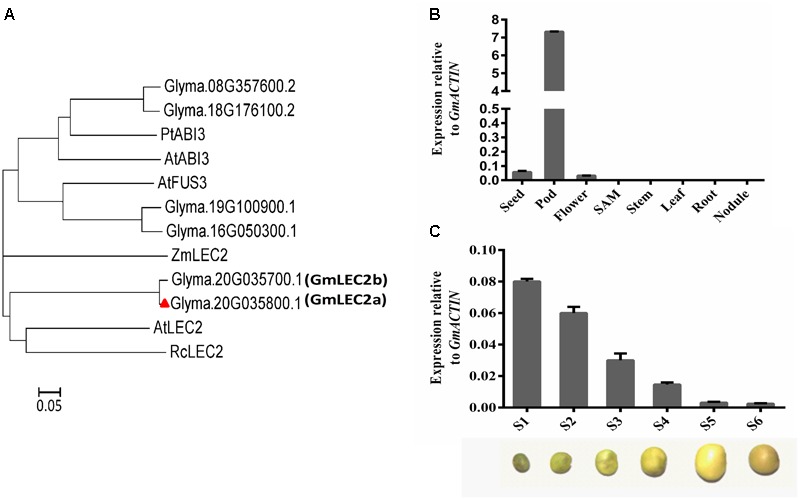
Identification of GmLEC2. **(A)** Phylogenetic analysis of GmLEC2s with other functionally characterized LEC2 proteins from Arabidopsis, castor bean, and maize. Rooted phylogenetic tree was constructed by using MEGA6 program through neighbor joining method. Bar shows 0.05 amino acid substitution. **(B)** Expression patterns of *GmLEC2a* in soybean tissues. Seeds, pods, nodules and flower from 8 to 11 weeks old plants, root, stem and leaf from 12 to 15 days old seedling were harvested for qRT-PCR. Transcript levels are expressed relative to that of *GmACTIN*. **(C)** Expression level of *GmLEC2a* in seed at six different developmental stages. Stages were classified on the basis of seed weight, as follows: stage 1 (S1). 40–70 mg; stage 2 (S2). 80–100 mg; stage 3 (S3).150–200 mg; stage 4 (Stage 4). 250–300 mg; stage 5 (S5). 350–430 mg; stage 6 (S6). 320–350 mg. Transcript levels are expressed relatively to that of *GmACTIN*.

To understand how the biosynthesis of soybean storage substances in seeds is regulated, we cloned soybean TFs that are homologs to these functionally characterized counterparts from Arabidopsis (**Figure 1A**). Homology search with Arabidopsis LEC2 protein against soybean genome identified two genes that share the highest homology with AtLEC2, GmLEC2a (Glyma.20G035800.1), and GmLEC2b (Glyma.20G035700.1). These two tandem duplicated genes on Chromosome 20 share more than 95% identity in protein sequence, and both are similarly and exclusively expressed in young embryos (flower and/or pod) (**Figure 1A** and Supplementary Figure S1). We thus chose one of them, GmLEC2a, for our functional study. GmLEC2a shared highest identity with other B3 domain TF LEC2 proteins from Arabidopsis, maize, and castor bean (Kim et al., 2014; Grimault et al., 2015). GmLEC2a shared 46% identity with castor bean LEC2, and 41% identity with Arabidopsis LEC2-like protein (**Figure 1A**).

Quantitative RT-PCR (qRT-PCR) data from eight different tissues of soybean plant indicated that *GmLEC2a* is predominantly expressed in the pod, seed, and flower (**Figure 1B**). The highest expression pattern of *GmLEC2a* in pod matched the expression patterns of *GmLEC2a* in public database (Supplementary Figure S1). Several studies indicated that LEC2 controls the embryogenesis and seed development (Meinke, 1994; Stone et al., 2008). To understand the role of GmLEC2 in seed development, the expression of *GmLEC2a* at different developmental stages of seeds was analyzed. Seed development was classified into six different stages on the basis of seed weight as described in our previous study (Chen et al., 2016). We found the highest *GmLEC2a* expression in seeds at the early stages of development which was then dropped along over the development of seed toward maturation (**Figure 1C**). The results are in agreement with the Arabidopsis *LEC2*, whose higher transcript level was detected in seeds at the pre-globular stage relative to seeds at mature stage (Fatihi et al., 2016). These results suggest that GmLEC2a could be a regulator of seed development in soybean.

### *GmLEC2a* Expression in *Arabidopsis thaliana* Alters Seed Oil Production and TAG Composition

**FIGURE 2:**
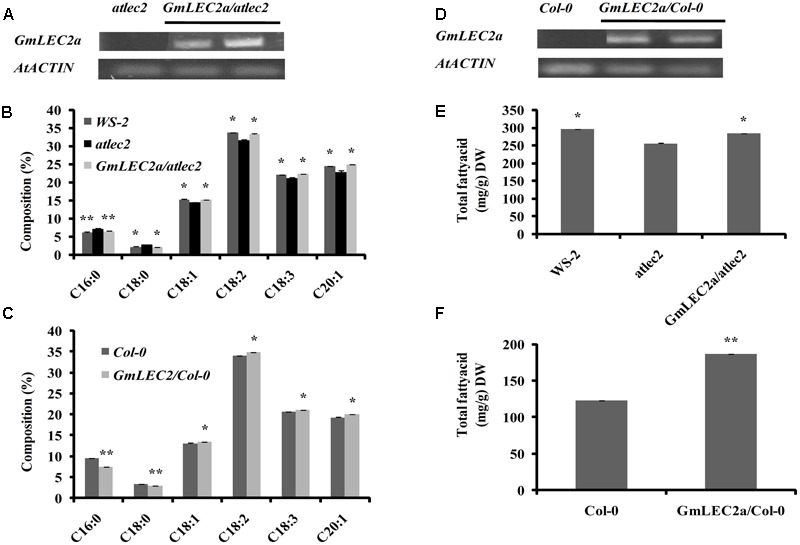
TAG content in *GmLEC2a* over-expression Arabidopsis seeds. Ectopic expression of GmLEC2a in Arabidopsis alters TAG composition and enhance TAG content in mature seeds. **(A)** Expression of *GmLEC2a* in *atlec2* mutant seeds by semi-qRT-PCR. **(B)** Composition of fatty acids in seeds of Arabidopsis *atlec2*, wild-type *WS-2*, and *GmLEC2/atlect2* complementation plants. **(C)** Composition of fatty acids in seeds of Col-0 and *GmLEC2aOE.* **(D)** Over-expression of *GmLEC2a* in Col-0 (*GmLEC2aOE*) detected by using semi-qRT-PCR. **(E**) TAG contents in seeds of Arabidopsis *atlec2*, wild-type *WS-2*, and *GmLEC2/atlect2* complementation plants. **(F)** TAG content in seeds of wild-type Col-0 and *GmLEC2a* overexpression (GmLEC2aOE) plants. Transcript levels are expressed relatively to that of *AtACTIN*. All data are three biological replicates and are expressed as means ± SD. ^∗∗^*P* < 0.01 and ^∗^*P* < 0.05 by Student’s *t*-test (*n* = 3). Asterisks indicate the significant difference relative to the *atlec2* mutant.

The chemical composition of seed is an important trait from agricultural perspectives. For example, the oil and starch storage in several crops like soybean, maize, and canola have received immense attention owing to their economical importance and potential applications in biofuels and various food products. To further test the function of GmLEC2, we performed genetic complementation by expressing *GmLEC2a* under the control of *CaMV35S* promoter in an Arabidopsis *atlec2* mutant. The homozygous *atlec2* mutant plants transformed with *Agrobacterium tumefaciens* harboring *pB2GW7-GmLEC2a* were screened for constitutive *GmLEC2a* expression (Supplementary Figure S2). The seeds from T3 plants of 15 independent lines expressing *GmLEC2a* as confirmed by qRT-PCR were used for TAG analysis (**Figure 2A**). The independent GmLEC2a/atlec2 transgenic lines rescued the wild-type FA composition. The GmLEC2a seeds showed high level of oleic acid (18:1), linoleic acid (18:2), linolenic acid (18:3), and eciosonic acid (20:1) in the TAG molecule relative to *atlec2* mutant (**Figure 2B**). The total seed FA analysis revealed a major change in content of 20:1, which was 8% higher in *GmLEC2a* complemented seeds as compared to *atlec2* seeds. A nearly 5 and 4% increment in 18:2 and 18:3 was noticed in *GmLEC2a*-transgenic lines, respectively. An average decrease of 28% in 18:0 (stearic acid) while a 10% reduction in 16:0 (palmitic acid) was observed in seeds from *GmLEC2a*-expressed plants compared to seeds from *atlec2* mutant plants. GmLEC2a expression increased 10% of the total TAG content in complemented Arabidopsis seeds relative to *atlec2* mutant seeds (**Figure 2E**).

To gain more insights into the function of GmLEC2a, *GmLEC2a* was over-expressed in Arabidopsis wild-type plants in Columbia-0 (Col-0) ecotype (**Figure 2D** and Supplementary Figure S3). The T3 seeds ectopically expressing *GmLEC2a* showed 34% more oil production than the Col-0 (**Figure 2F**). Compared to wild-type seeds, a 26 and 12% reduction in palmitic acid and stearic acid contents was recorded, respectively, in transgenic seeds. The 18:1 FA content in GmLEC2a-seeds increased by 3% compared to control (wild-type seeds). The contents of each 18:2 and 18:3 FAs were increased by 2% in *GmLEC2a*-expression seeds. The level of 20:1 FA in *GmLEC2a*-transgenic lines was 4% higher than wild- type mature seeds (**Figure 2C**).

### *GmLEC2a* Genetically Complements *atlec2* Mutant Phenotypes

**FIGURE 3:**
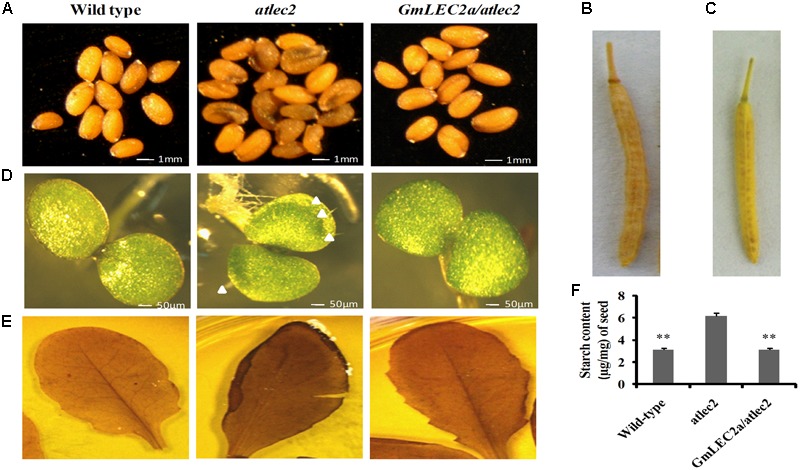
*GmLEC2a* genetically complements *AtLEC2.* **(A)** *atlec2* seeds contain more phenolic compounds relative to the wild-type and *GmLEC2a/atlec2* complemented seeds. **(B)** The recovered mature silique length (13 mm) in *GmLEC2a/atlec2* complementation plants. **(C)** The shorter mature silique of *atlec2* (∼11 mm) relative to *GmLEC2a*/*atlec2* complementation plants. **(D)** The abnormal trichomes on adaxial cotyledon surface of *atlec2* mutant were restored by expression of *GmLEC2a* in *atlec2* (*GmLEC2a*/*atlec2* complementation). The wild-type, *atlec2*, and complementation plant seeds were grown on MS media and 4-days old seedlings were photographed. Arrows highlight the trichomes. **(E)** I-KI Staining of leaf starch, the *lec2* mutant leaves were darker when stained with iodine than the wild-type (*WS-2*) and complementation plant (*GmLEC2a/atlec2*) leaves indicating high starch content in the leaves.**(F)** Starch content in *WS-2, atlec2*, and complementation plant *GmLEC2a/atlec2* seeds. The bar represents average of 5 transgenic or control lines. All data are three biological replicates and are expressed as means ± SD. ^∗∗^*P* < 0.01 and ^∗^*P* < 0.05 by Student’s *t*-test (*n* = 3). Asterisks indicate the significant difference relative to the *atlec2* mutant.

The role of GmLEC2a in plant morphology and seed development was investigated. For this purpose, pods and seeds from mutant *atlec2*, complementation (*GmLEC2a/atlec2*), and wild-type plants grown in the identical environmental conditions were examined for pod length and seed color. The *GmLEC2a* expression changed the color and size of *atlec2* seeds (**Figure 3A**). When compared to *GmLEC2a* expressed mutant seeds, *atlec2* seeds were dark in color. The dark color represents the accumulation of phenolic compounds in the mutant seeds (Angeles-Núñez and Tiessen, 2011). The GmLEC2a increased the length of siliques in genetically complemented *atlec2* mutant plants (**Figures 3B,C**). The average pods length from *GmLEC2a/atlec2* plants were between 13 and 14 mm (**Figure 3B**), which is longer than those of the pods from mutant *atlec2* plants (∼11 mm) (**Figure 3C**).

To elucidate the effect of GmLEC2a on cotyledon morphology, the green mature seeds before desiccation were collected from *atlec2, GmLEC2a*-complementation, and wild-type Arabidopsis plants. The seeds were surface sterilized and germinated on MS media. The 4-day old cotyledons were photographed using the microscope Olympus SZX16 for the presence of trichome. While the cotyledons from the mutant bear trichomes, which is a vegetative leaf characteristic, the *GmLEC2a*-expression in *atlec2* mutant seeds restored normal cotyledons with no trichomes on the adaxial surface (**Figure 3D**).

### *GmLEC2a* Expression Alters the Starch Concentration of *atlec2* Seeds and Leaves

In developing seeds, starch metabolism usually behaves reciprocally with the oil storage (Angeles-Núñez and Tiessen, 2011). The Arabidopsis mature seeds contain less starch and high oil and protein content. To investigate the role of GmLEC2 in starch accumulation in Arabidopsis vegetative tissues and seeds, we performed iodine staining. The leaves from 4 week old *GmLEC2a/atlec2, atlec2* and wild-type plants were treated with iodine solution in order to stain and observe the starch granules. The stronger starch-iodine staining of *atlec2* relative to wild-type and *GmLEC2a/atlec2* leaves showed high accumulation of starch in the mutant leaves (**Figure 3E**). Furthermore, the reduced starch concentration in mature Arabidopsis seeds over-expressing *GmLEC2a* was detected compared to *atlec2* seeds (**Figure 3F**). The reduced starch content in seeds expressing *GmLEC2a* can be correlated with the higher seed TAG contents.

### Ectopic Expression of *GmLEC2a* in Soybean Hairy Roots Promoted TAG Biosynthesis

**FIGURE 4:**
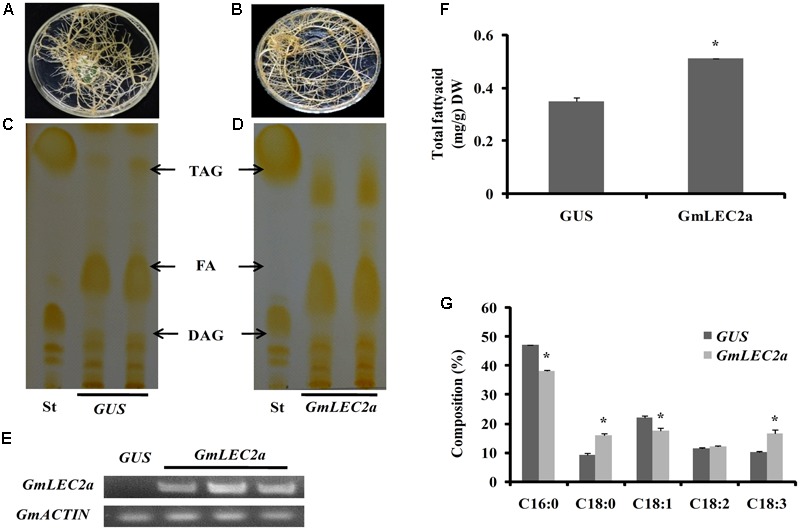
Ectopic expression of *GmLEC2a* in soybean hairy roots. **(A,B)** Hairy roots over-expressing *GmLEC2a* and *GUS.* The cotyledons of germinating soybean seeds were used for infection with *Agrobacteria* K599 harboring p*B2GW7-GmLEC2a or GUS* gene (control). *The g*enerated hairy roots over-expressing *GUS* **(A)** and *GmLEC2a* **(B)** were selected on MS medium containing ppt. **(C,D)** TLC analysis of neutral lipids extracted from hairy root over-expressing *GmLEC2a* **(D)** and *GUS* control **(C)**. **(E)** qRT-PCR conformation of *GmLEC2a* or *GUS* gene expression. Transcript levels are expressed relatively to that of *GmACTIN.* **(F,G)** Comparison of TAG composition **(G)** and content **(F)** in hairy roots over-expressing *GmLEC2a* and *GUS* control grown and extracted under the identical conditions. More than 5 independent transgenic hairy root lines were analyzed. Data are from three biological replicates. All data are three biological replicates and are expressed as means ± SD. ^∗∗^*P* < 0.01 and ^∗^*P* < 0.05 by Student’s *t*-test (*n* = 3).

To further understand the role of GmLEC2a in oil production, we expressed *GmLEC2a*, driven by a CaMV35S promoter, in soybean hairy roots derived from cotyledons because transformation of soybean plant is still a main obstacle with low regeneration and positive rates. The hairy roots transformation is a convenient approach to verify soybean genes function instead of time consuming and lower transformation rate of soybean plants (Chen et al., 2016). The hairy roots expressing *GUS gene* under the 35S promoter was used as a control (**Figure 4A**) to investigate how significantly GmLEC2a effects the chemical composition of transgenic hairy roots (**Figure 4B**). The ectopic expression of *GmLEC2a* was confirmed with semi-quantitative RT-PCR and qRT-PCR (**Figure 4E** and Supplementary Figure S4); and TAG contents were confirmed with TLC analysis (**Figures 4C,D**) in combination with gas chromatography (GC) measurement (**Figures 4F,G**). More than 10 independent hairy root lines were analyzed to check the GmLEC2a over expression function in TAG accumulation. The total TAG in *GmLEC2a-*hairy roots increased by 31.5% on average compared to *GUS* control (**Figure 4F**). The sharp FA bands shown on preparative TLC plates can be attributed to the hydrolysis of lipids during extraction process, rather than endogenous FAs (Chen et al., 2016). The high TAG accumulation in *GmLEC2a-*overexpressed hairy roots indicated that GmLEC2a either triggered TAG biosynthesis or TAG accumulation. Further, the FA composition analysis in these TAGs suggested that *GmLEC2a* expression in soybean hairy roots preferred to synthesize TAGs with a significant amount of α-linolenic acid (18:3) acyl-chains (**Figure 4G**). A marked increase of 38 % in 18:3 was detected whereas 18:2 amount increased by 5% in the transgenic roots. Meanwhile, a marginal decrease in 16:0 and 18:1 were observed. The portion of 18:0 in *GmLEC2a*-transgenic roots was increased by 40% on average relative to *GUS* control (**Figure 4G**).

### *GmLEC2a* Over-Expression Alters the Total Protein Level in Hairy Roots and Seeds

Soybean seeds contain more protein content than oil as a major storage substance. Therefore, in order to investigate how GmLEC2a regulates the metabolic relationship between total proteins and oils, we extracted and measured the total proteins in over-expressing *GmLEC2a* soybean hairy roots and Arabidopsis seeds. The amount of total proteins was less in *GmLEC2a*-hairy roots as compared to the control hairy roots (Supplementary Figure S5A). The GmLEC2a significantly increased the protein level in transgenic Arabidopsis seeds (Supplementary Figures S5B,C). The *atlec2* mutant seeds possess 39% less protein content than the wild-type and *GmLEC2a/atlec2* Arabidopsis seeds. Further in GmLEC2a expressed wild-type (Col-0) seeds an increase of 7.4% in total protein level was recorded.

### Transcriptome Analysis of *GmLEC2a-*Overexpressing Soybean Hairy Roots Revealed Its Regulatory Targets

**FIGURE 5:**
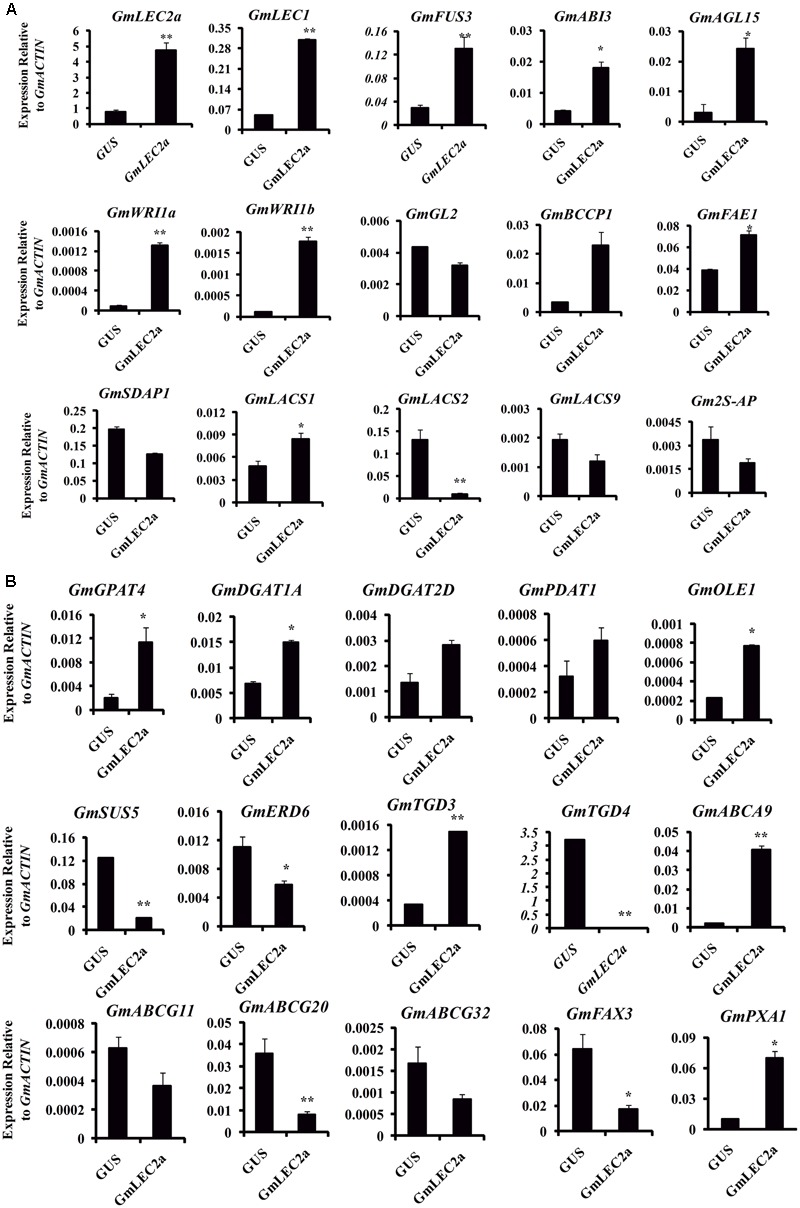
Validation of transcriptomic data with qRT-PCR. **(A)** Verification of transcription factors and few transporters transcriptomic data with qRT-PCR. **(B)** Verification of transcriptomic data of genes involved in TAG, fatty acid, sucrose biosynthesis and transporters of fatty acid, wax with qRT-PCR. Transcript levels are expressed relatively to that of *GmACTIN*. All data are three biological replicates and are expressed as means ± SD. ^∗∗^*P* < 0.01 and ^∗^*P* < 0.05 by Student’s *t*-test (*n* = 3).

To further dissect the regulatory network of GmLEC2a in accumulation of storage substances, transcriptome profiling on *GmLEC2a*-overexpressing soybean hairy roots was performed by using RNA sequencing (RNA-Seq) technology. The data from RNA-Seq experiments mapped to soybean genome was analyzed for differential gene expression (DGE) using the DESeq (2012) R package. The analysis of Gene Ontology (GO) term enrichment indicated that the DEGs were involved in many biological processes, such as lipid biosynthesis, embryogenesis, seed maturation, and carbohydrate metabolic processes. To verify the results, few genes were selected for validation with qRT-PCR (**Figures 5A,B**).

In plants, the biosynthesis and storage of seed storage substances, such as TAG, starch, and proteins involve many metabolic enzymes and regulatory factors (Angeles-Núñez and Tiessen, 2011). These complex physiological processes are highly coordinated in terms of enzymes transporters, and are regulated at transcriptional levels with the seed development factors by various TFs (Fatihi et al., 2016; Manan et al., 2016). Our results indicate that transcripts of metabolic genes involved in FA and TAG biosynthesis were markedly altered in *GmLEC2a*-hairy roots compared to the *GUS* control. *GmLEC2a* over-expression up-regulated several downstream TFs, such as *FUS3, ABI3, LEC1*, and *Dof11* (**Table 1**). Many plastidic FA biosynthesis genes, such as KAS and KCS, etc. were also up-regulated (Supplementary Table S2). Many ER-localized TAG biosynthetic genes, such as GPAT, DGAT, and PDAT were also up-regulated (Supplementary Table S2). However, few lipid transporters (**Table 2**) and sucrose synthases (**Table 3**) were negatively regulated by GmLEC2a. The genes encoding enzymes involved in amino acid and protein biosynthesis were differentially regulated by over-expression of *GmLEC2a* in soybean hairy roots (**Table 4**). In the light of these results, we can conclude that GmLEC2a has potential function in controlling the metabolism of storage substances in developing soybean seeds.

**Table 1:** List of transcription factors regulated by *GmLEC2a* in TAG biosynthesis pathway.

Glyma ID	GUS	GmLEC2	Log_2_ FC	Gene description
GLYMA02G33090	7.42	33.96	2.19	Wrinkle1
GLYMA07G02380	9.54	19.81	1.05	Wrinkle1
GLYMA13G40420	3.77	14.84	1.97	Dof11
GLYMA19G27336	37.73	82.68	1.13	FUS3-like
GLYMA08G47240	125.46	257.92	1.04	ABI3-like
GLYMA07G39820	1.88	5.30	1.49	LEC1

**Table 2:** List of transporters mediated by GmLEC2a in lipid biosynthesis.

Glyma ID	GUS	GmLEC2a	Log_2_ FC	Gene description
GLYMA18G47600	103.77	244.87	1.23	TGD3-like
GLYMA09G41100	154.71	36.04	-2.10	FAX 3 like
GLYMA09G05660	54.714	1.06	-5.68	TGD4-like
GLYMA05G08100	81.13	34.98	-1.21	ABCG32a -like
GLYMA17G12910	13.21	5.30	-1.31	ABCG32b-like
GLYMA13G07890	5.66	2.12	-1.41	ABCG11-like
GLYMA19G35970	174.52	57.24	-1.60	ABCG20-like
GLYMA06G20360	229.23	1513.75	2.72	ABCA9a-like
GLYMA04G34140	6.60	23.32	1.82	ABCA9b-like
GLYMA03G38000	10.37	25.44	1.29	LACS1-like
GLYMA07G20860	278.29	110.25	-1.33	LACS2a-like
GLYMA20G01060	416.96	157.95	-1.40	LACS2b-like
GLYMA12G05140	103.77	36.04	-1.52	LACS2c-like
GLYMA06G11860	26.41	7.42	-1.83	LACS9-like

**Table 3:** List of sucrose synthase genes differentially expressed in *GmLEC2a* over-expressed roots.

Glyma ID	GUS	GmLEC2a	Log_2_ FC	Gene description
GLYMA16G34290	5.66	1.06	-2.41	Sucrose synthase 7A
GLYMA02G40740	11.32	4.24	-1.41	Sucrose synthase 7B
GLYMA11G33240	5.66	1.06	-2.41	Sucrose synthase 5A
GLYMA14G03300	120.74	11.66	-3.37	Sucrose synthase 5B
GLYMA14G08070	30.18	4.24	-2.83	ERD6-like

**Table 4:** Differentially regulated genes by GmLEC2a encoding enzymes of amino acid and protein biosynthesis.

Glyma ID	GUS	GmLEC2a	Log_2_FC	Gene description
GLYMA19G05580	3.77	30.74	3.02	Proline dehydrogenase 2A
GLYMA18G51400	195.27	1267.82	2.69	Proline dehydrogenase 2B
GLYMA19G05570	46.22	295.75	2.67	Proline dehydrogenase 2C
GLYMA06G00990	1168.81	4529.60	1.95	Arginine decarboxylase
GLYMA16G26940	2.83	23.32	3.04	Glutamate dehydrogenase 1A
GLYMA16G26940	2.83	23.32	3.04	Glutamate dehydrogenase 1B
GLYMA02G07940	33.02	143.11	2.12	Glutamate dehydrogenase1C
GLYMA05G05460	20.75	44.52	1.10	Glutamate dehydrogenase1D
GLYMA13G28180	455.64	138.86	-1.71	Glutamine synthase
GLYMA15G10890	709.39	346.63	-1.03	Glutamine synthetase
GLYMA14G37440	47.17	240.63	2.35	Asparagine synthetase
GLYMA10G39150	0	8.48	Inf	Beta-conglycinin A
GLYMA03G32030	0	7.42	Inf	Glycinin G1
GLYMA10G04280	0	9.54	Inf	Glycinin G4
GLYMA13G18450	0	2.12	Inf	Glycinin
GLYMA01G17820	30.19	1.060	-4.83	2S albumin protein (2S-AP)
GLYMA16G26480	48.11	11.66	-2.04	2S albumin protein

## Discussion

### *GmLEC2a* Is the Soybean Ortholog of LEC2 from Other Plants

The genetic complementation and over-expression analysis in Arabidopsis and hairy roots indicated that *GmLEC2a* is the soybean ortholog of LEC2 from model and other oilseed crops. GmLEC2a restored the defective phenotypes of *atlec2* in terms of plant morphology and oil biosynthesis. In addition, starch and protein concentrations of *atlec2* plants were appreciably rescued in *GmLEC2a/atlec2* plants. The total TAG content of *atlec2* seeds was partially rescued in *atlec2* seeds to the level of wild-type. The increase in total TAG content of atlec2 seeds was 10% that is lower than the TAG content of wild-type seeds. The use of 35S-promoter could be the result of different level of *atlec2* complementation in different parts of plant. Although one of the most common promoters used for plant genetic engineering is CaMV35S. The expression profile study suggests that it is not fully expressed in all tissues and cell types (Abbasi et al., 2010). The higher TAG content and altered FA composition in GmLEC2a-expressed Arabidopsis seeds indicated that GmLEC2a shares a similar function with RcLEC2 in TAG modification and accumulation (Kim et al., 2014). This study is in line with reports by Grimault et al. (2015) suggesting that LEC2 function is partially diverged in crops.

A recent study on regulatory mechanism for soybean oil biosynthesis characterized the functions of GmFUS3 and GmABI3 through Arabidopsis transformation and ectopic expression in soybean hairy roots (Zhang et al., 2017). However, the *GmLEC2a* was shown to be non-functional and not expressed in soybean tissues, which is contradictory to our current study (Zhang et al., 2017). We here showed that not only *GmLEC2a* and *GmLEC2b* are expressed exclusively in young embryos and early seed developmental stages, but also GmLEC2a functionally rescued Arabidopsis counterpart mutant’s phenotypes.

### Key Regulatory Genes in *GmLEC2a* Transgenic Hairy Roots Were Up-regulated

The modified FA composition and TAG accumulation of ectopically expressed soybean hairy roots and Arabidopsis seeds is the result of activation of complex regulatory network of FA biosynthesis. LEC2 protein induces the expression of other TF genes such as *FUS3* and *ABI3* that are key regulators of lipid metabolism, embryogenesis, seed development, and maturation (Mu et al., 2008; Baud et al., 2009). The GmLEC2a-mediated expression of other TFs involved in TAG biosynthesis and is listed in **Table 1**. The expression of *GmLEC1, GmFUS3*, and *GmABI3* were moderately higher in *GmLEC2a*-transgenic hairy roots. The enhanced expression of LAFL (LEC1/AFL) clade can be associated with high TAG accumulation in transgenic soybean roots. *GmLEC2a* transgenic roots contain higher level of *GmDof11.* Over-expression of *GmDof11* increased the total FA content and seed yield in Arabidopsis by activating ACCase (acetyl-CoA carboxylase) and LACS (Long chain acyl synthase) activity (Wang et al., 2007). WRI1, directly activates expression of metabolic genes encoding multiple enzymes of FA synthesis and late glycolysis, is a direct target of LEC2 (Baud et al., 2009). We therefore analyzed the expression of WRI1 in the transgenic roots. The *GmLEC2a* over-expression up-regulates homologs of Gm*WRI1* (**Table 1**) which may further mediate up-regulation of TAG biosynthetic genes. The homology search revealed that GmLAFL, GmWRI1, and GmDof11 are the soybean orthologs of LAFL, WRI1, and Dof from other crops (Supplementary Figure S7).

### Key Genes Involved in Plastidic FA Biosynthesis Are Up-regulated in *GmLEC2a*-Transgenic Roots

**FIGURE 6:**
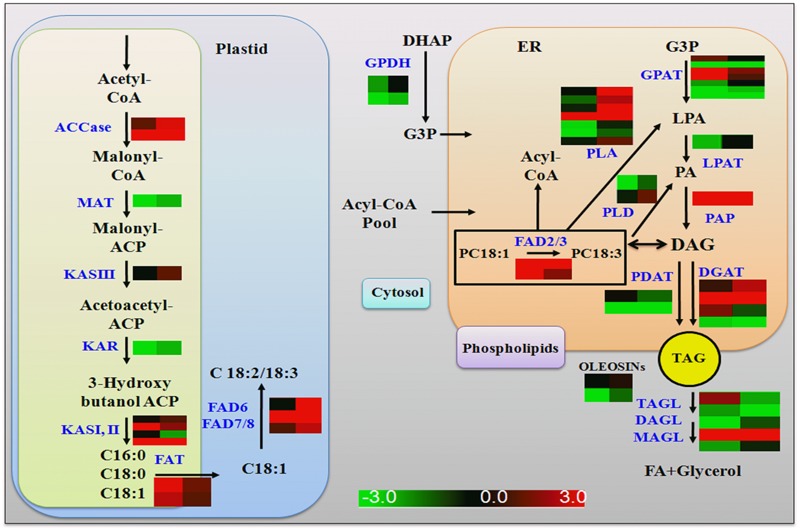
Summary of genes regulated by GmLEC2a in *de novo* TAG biosynthesis pathway. ACCase, Acetyl-CoA carboxylase; MAT, malonyl-CoA-ACP transacylase; KAS, 3-oxoacyl ACP synthase; KAR, 3-oxoacyl ACP reductase; FATA/B, fatty acyl-ACP thioesterase A/B; FAD2/6, D12(ω6)-Desaturase; FAD3/7/8, D15 (ω3)-Desaturase; GPAT, glycerol-3-phosphate acyltransferase; LPAT, lysophosphatidyl acyltransferase; PP, phosphatidate phosphatase DGAT, diacylglycerol *O*-acyltransferase; PDAT, phospholipid: diacylglycerol acyltransferase; PLA1/2, phospholipase A1/2; PLD, phospholipase D. Right panel of heat map represents gene expression in *GmLEC2a-* transgenic roots; left panel represents expression in control (*GUS*) hairy toots.

FAs are precursors for all lipids, whether they serve as energy storage or membrane structure. In chloroplast, after ACCase generated malonyl CoA, the FA synthase complex transfers malonyl moiety to acyl-carrier proteins (ACPs) for the generation of long-chain FAs, mainly C16:0, C18:0 and C18:1 (Manan et al., 2016). In transgenic soybean roots, the components of FAs synthase complex (KASIII, KAR, MAT) were up-regulated in *GmLEC2a* expressed hairy roots compared to control. An overview of enzymes associated with FA and TAG biosynthesis pathway with altered expression are shown in **Figure 6** and summarized in Supplementary Table S2. The FA desaturases (FADs) control the FA composition in the total lipids. In lipid biosynthesis pathway, conversion of oleic acid to linolenic acid is carried out by FAD2 in ER while FAD6 catalyzes oleic to linolenic acid conversion in plastid. The FAD7/FAD8 converts linolenic acid to α-linolenic acid in plastids while in ER this reaction is carried out by FAD3 (Singer et al., 2014). The transcriptome data showed that only *GmFAD7* and *GmFAD8* (Supplementary Table S2 and Figure S8) were significantly up-regulated whereas the transcripts of *GmFAD3* homologs were reduced in *GmLEC2a* transgenic hairy roots. The TAG from transgenic roots contains more linolenic acid level (**Figure 4G**), that verified the transcriptome data. Arabidopsis FATTY ACID ELONGATION 1 (AtFAE1) is responsible for FA chain elongation from C18 to C20 and is considered a target of AtFUS3 and AtABI3 but not AtLEC2 (Roscoe et al., 2015). The *fae1* mutation blocks the C18:1 to C20:1 conversion (Trenkamp et al., 2004). TAG content in *GmLEC2a*-expressed Arabidopsis seeds also possesses high proportions of C18:3 and C20:1 FAs (**Figures 2B,C**). GmLEC2a over-expression in hairy roots enhanced the expression of *AtFAE1* homologs (GLYMA04G20620, GLYMA06G24480). Our data verified that GmLEC2a could regulate the GmFAE1 expression for the production of long chain FAs.

### Genes Involved in the ER-TAG Biosynthesis Are Up-regulated in *GmLEC2a*-Transgenic Roots

Glycerol-3-phosphate acyltransferases (GPATs) catalyzes the glycerol-3-phosphate into lysophosphatidic acid (LPA), a first step of TAG and phospholipid synthesis (Chapman and Ohlrogge, 2012). The *BnGPAT4* expression in *gpat4* mutant background altered the leaf cutin and stomata structure (Chen et al., 2011). LPA is further acylated at the sn-2 position into PA by LPA acyltransferase (LPAAT) (Yang et al., 2010). PA is positioned in the center of lipid biosynthesis of TAG, with turnover of phospholipids, and lipid metabolism in the ER (Bates and Browse, 2012). PA transport to various locations and their regulation are essential for plant growth and oil production (Allen et al., 2015). DGAT1 is a major enzyme that catalyzes last step in TAG synthesis (Zhang et al., 2009). Seed specific expression of *DGATs* leads to high TAG deposition and increased seed weight compared to control plant (Jako, 2001). Up-regulation of *DGATs* in soybean hairy roots results in elevated oil deposition and alters the hormone level (Chen et al., 2016). In addition to DGAT1, PHOSPHOLIPID, DIACYLGLYCEROL ACYLTRANSFERASE1 (PDAT1), which catalyzes the acyl-CoA-independent synthesis of TAG, also contributes to seed oil biosynthesis in Arabidopsis (Zhang et al., 2009). The fold increase in gene transcripts encoding GmGPAT, GmLPAT, GmPAP, GmDGAT, and GmPDAT enzymes by GmLEC2a over-expression in hairy roots is shown in Supplementary Table S2 and **Figure 6**.

A previous study showed that LEC2 binds with the two RY elements present in the promoter regions of *OLEOSIN* genes (Kroj et al., 2003). Another study revealed that neighboring RY elements respond efficiently to LEC2 activation of *OLEOSINs* expression (Che et al., 2009). The *AtLEC2* and its ortholog from *Ricinus communis* (*RcLEC2*) promoted transcription of five seed specific *OLEOSIN* genes in its leaves (Kim et al., 2014). The GmLEC2a protein in soybean influences the GmOLE1 (*OLEOSIN1)* protein (**Figure 5B** and Supplementary Table S2). In the current study, 10 target genes of GmLEC2a identified in the transcriptome analysis of soybean roots were selected to find the RY elements in their promoter regions (Supplementary Figure S6). The presence of RY elements in the upstream region provided the clue that the selected genes could be direct targets of *GmLEC2a*. It is proposed that plastidic FA and ER TAG biosynthesis genes are most likely indirectly up-regulated by GmLEC2a, however, through activation of GmLEC2a-mediated up-regulation of GmWRI1, GmDof11, and GmFUS3. Nevertheless, GmLEC2a could directly up-regulate GmOLE1.

### Genes Involved in the TAG Catabolism and Lipid Hydrolysis Are Regulated by *GmLEC2a*

During germination, TAGs are hydrolyzed into FAs and glycerol backbone to provide energy to the growing seedling. Analysis of *sdp1* mutant shows that SDP1 (Sugar-Dependent 1) is majorly responsible for TAG breakdown subsequent to seed germination (Kelly et al., 2011; Fan et al., 2014). The enzymes involved in the degradation of various phospholipids, such as phospholipase A, C, and D (PLA, PLC, and PLD) have been extensively studied in plants (Zhao, 2015). For instance, research indicates that PLA, C, or D-mediated phospholipid hydrolysis and generated PA, DAG, lysophospholipids, and α-linolenic acid plays various roles in lipid metabolism, such as TAG biosynthesis and acyl editing, plant response to abiotic and biotic stresses, and cellular dynamics (Zhao, 2015). The GmLEC2a activated the phospholipases whereas the TAG and DAG lipases were deactivated (Supplementary Table S2 and Figure S8) that could be the reason for high TAG accumulation and modified FA composition in transgenic soybean roots and Arabidopsis seeds.

### GmLEC2a Controls the Regulation of Lipid Transporters

**FIGURE 7:**
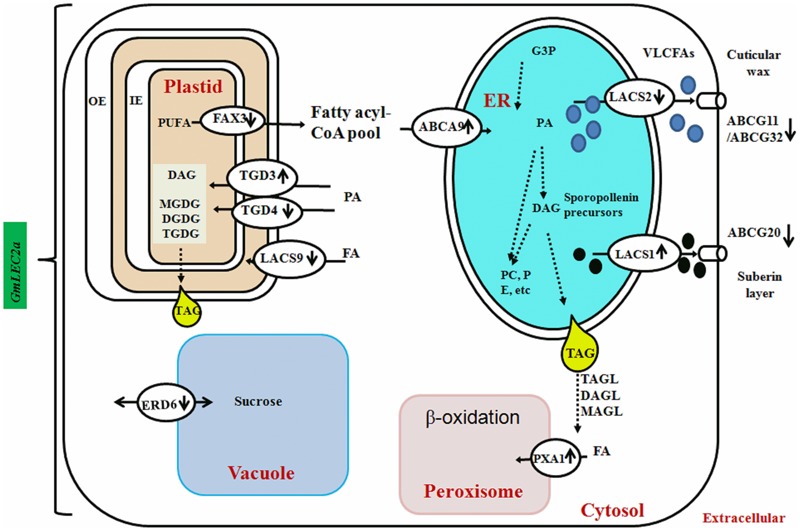
GmLEC2a control the lipid and sucrose transport in the soybean. FAs synthesized in chloroplasts need to be exported to the ER for the biosynthesis of TAG, polar lipids, wax and cuticle. Fatty acid export1 (FAX1) localized in the inner envelope (IE) exports FAs out of chloroplast lumen. TGDs transport complex and LACS9 present at chloroplast envelope membranes is involved in import of phospholipids and other glycolipid precursors into the chloroplast. The ABCA9 is responsible for FA import into ER for synthesis of TAG and phospholipids. The ER assembled precursors for cutin and wax biosynthesis are transported out by LACS1, LACS2. At the plasma membrane ABCG11, ABCG32 export wax and cutin precursors out of epidermal cells for deposition on plant tissue surfaces. The ABCG20 provide precursors for suberin layer formation. The PXA1 transport FA into peroxisome for β-oxidation. The *AtERD6*–like encode a sucrose transporter that is similar to sugar beet tonoplast membrane protein. The direction of vertical arrows beside the name of transporter indicated that specific transporter is either down or up-regulated by GmLEC2a.

FAs synthesized in chloroplasts are transported into the cytosol to form cytosolic acyl-CoA pools, which are subsequently transported to the ER for assimilation into membrane structure phospholipids and storage neutral lipids, DAG or TAGs (Chapman and Ohlrogge, 2012). Arabidopsis ABC lipid transporters are believed to be involved in the ER-chloroplast phospholipid trafficking consisting of trigalactosyldiacylglycerol (TGD) 1, 2, and 3, which are located in the inner membrane envelope. TGD4 is predicted to be a barrel protein that resides in outer chloroplast membrane involved in lipid precursor trafficking from ER to chloroplast (Hurlock et al., 2014; Fan et al., 2015). The fatty acid exporters (FAX) are supposed to be involved in FA export from plastid to cytosol (Li et al., 2015). Among them, FAX1; located at inner chloroplast membrane is functionally characterized as exporter of FAs from plastid to cytosol (Li et al., 2015). AtABCA9, an Arabidopsis ER-localized A-type ABC transporter, was revealed to import FA from cytoplasm to ER (Kim et al., 2013). AtLACS1 and AtLACS2 facilitate the uptake of VLCFAs though AtLACS9 is characterized for transport of ER-derived FAs into chloroplast (Manan et al., 2016). The transcriptomic analysis of FA transporters revealed that GmTGD3, GmLACS1, and GmABCA9 were up-regulated in *GmLEC2a*-transgenic roots (**Table 2** and **Figure 7**). While other transporters such as GmFAX3, GmTGD4 for FA transport, and GmABCG transporters required for sporopollenin and wax precursors transport from ER to extracellular surface were down-regulated in transgenic roots (**Table 2**), which were validated through qRT-PCR (**Figure 5B**). An overview of all FA, lipid, and sugar transporters regulated by GmLEC2a is shown in **Figure 7**. Phylogenetic tree was generated to show GmTGDs, GmFAXs, GmLACSs, and GmABCGs are true homologs of Arabidopsis and other crops (Supplementary Figure S9). Further, the relationship between already known members of ABC family (ABCA, ABCD, and ABCG) involved in lipid transport is shown in soybean through phylogenetic tree (Supplementary Figure S10). Moreover, the Arabidopsis LACS homologs were queried to search their homologs in *Glycine max* (Supplementary Figure S11).

### GmLEC2a Mediated Regulation of Starch Metabolism

The starch metabolism and turnover in developing and mature seeds is a complex pathway (Andriotis et al., 2016). During early developmental stages in Arabidopsis seeds, starch is transiently accumulated; however, very low amount remains in mature dry seeds. Several studies reported that seed starch level behaves reciprocally with the other main storage compounds such as oil and protein (Angeles-Núñez and Tiessen, 2010, 2011). Another study reported that starch accumulation is caused by the absence of certain metabolic enzymes (Andriotis et al., 2016). The assumption of metabolic competition between oil and starch biosynthesis pathways is further supported by *atwri1* (Focks and Benning, 1998) and *atlec2* (Angeles-Núñez and Tiessen, 2011) with elevated starch and reduced oil contents. The *sus2* and *sus3* silencing in Arabidopsis reduces 30–70% starch content while lipid content increases up to 55% in the mutant seeds (Angeles-Núñez and Tiessen, 2010). The deficiency of SUS enzymes transfers hexose-P channeling toward oil biosynthesis but away from starch during early period of seed development (Angeles-Núñez and Tiessen, 2010, 2011).

Studies have revealed that starch metabolic enzymes are spatially and temporally regulated by LEC2, FUS3, and ABI3 in developing seeds (Roscoe et al., 2015; Andriotis et al., 2016; Devic and Roscoe, 2016). The LEC2a-mediated transcriptional regulation of *SUS* genes is important for metabolite homeostasis. It is hypothesized that more starch in *atlec2* mutant could be the result of poor starch degradation during late developmental stages (Angeles-Núñez and Tiessen, 2011). To check the effect of GmLEC2a on SUS genes in soybean, we have analyzed SUS5 transcripts in GmLEC2a over-expressed hairy roots using qRT (**Figure 5B**). *GmLEC2a* down-regulated four homologs of *GmSUS* genes in soybean (**Table 3** and Supplementary Figure S12). It was speculated that the effects of GmLEC2a on starch and oil metabolism could be partially accounted by the fact that it controls GmWRI1 expression. W*RI1* loss has major effects on sugar, starch, protein, and oil metabolism (Focks and Benning, 1998). Arabidopsis *ERD6* (*Early response to dehydration*) gene encodes a putative sugar transporter and is considered to be localized in plant cell vacuole. The AtERD6 is considered similar as the sugar beet transporter, although the substrate specificity is not yet identified (Chiou and Bush, 1996; Kiyosue et al., 1998). The expression of *AtERD6* homolog was reduced by two-folds in *GmLEC2a*-transgenic hairy roots as compared to the control (**Table 3**).

### GmLEC2a Mediated Amino Acid and Protein Metabolism

The citric acid or TCA cycle, a central pathway, is composed of eight different reactions that occur in mitochondrial matrix along with the oxidation of pyruvate, which takes place in cytosol. For amino acids synthesis, TCA cycle provides oxaloacetate and 2-oxoglutarate as precursors (Rossignol et al., 2004). The conversion of alpha-ketogultarate into glutamate and vice versa is catalyzed with glutamate dehydrogenase (GDH1 and 2). The cycle ends at the production of oxaloacetic acid (oxaloacetate) that provides precursors for production of asparagine and asparatate amino acids. Asparatate is further catabolized into cysteine, threonine, and methionine (Ljungdahl and Daignan-Fornier, 2012). The soybean contains two major seed specific storage proteins 7S (β-conglycinin) and 11S (glycinin). These proteins constitute about 70% of the total storage proteins of seed. Soybean makes large adjustments during seed filling and maintains total protein content of the seed. If some major proteins are repressed, it compensates the loss by accumulating minor proteins (Schmidt et al., 2011).

**FIGURE 8:**
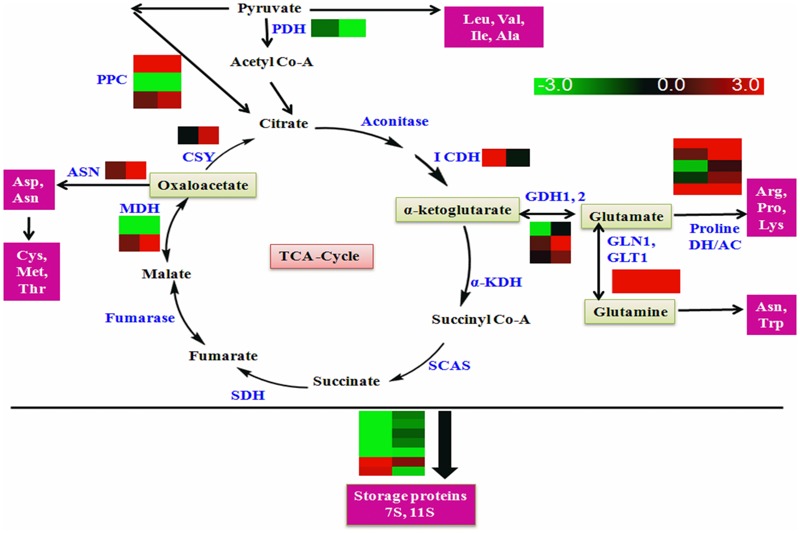
Schematic diagram of amino acid metabolism. PDH, pyruvate dedydrogenase; KDH, ketoglutarate dehydrogenase; ICDH, isocitrate dehydrogenase; SCAS, succinic Co-A synthase; PPC, pyruvate carboxylase; SDH, succinic dehydrogenase; MDH, malanate dehydogenase; CSY, citrate synthase GDH1, 2, glutamate dehydrogenase 1,2; GLN1, glutamine synthase; GLT1, glutamine synthetase, Proline DH, proline dehydrogenase; AC, arginine decarboxylase; ASN, asparagine synthase. Enzymes (Blue text); Amino acid precursors (Green box); Amino acids and proteins (pink box). The universal three letter code was used for amino acid representation. Right panel of heat map represents gene expression in *GmLEC2a*-transgenic roots, left panel represents expression in control (*GUS*) hairy toots.

Several enzymes of amino acid metabolism and storage protein synthesis were found to be regulated by GmLEC2a, as summarized in Supplementary Table S3 (amino acids), **Table 4** (proteins) and shown in **Figure 8**. The altered expression of genes encoding glutamate dehydrogenases was observed in *GmLEC2a* over-expressed hairy roots. Besides dehydrogenases, several other genes of amino acid metabolic pathway such as asparagine synthetase and glutamine synthetase, etc. were affected by *GmLEC2a* expression. An elevated expression of mRNA transcripts encoding 7S and 11S storage proteins was observed whereas 2S albumin proteins were reduced in transcriptome data (**Table 4**). In both GmLEC2a-expressed wild-type and *atlec2* mutant Arabidopsis seeds, the amount of total protein was higher compared to control, which is in accordance with AtlEC2 function. The *atlec2* mutation in Arabidopsis reduces 15% of total protein content of seed (Angeles-Núñez and Tiessen, 2011). However, in transgenic hairy roots the total protein content was reduced. The current study indicates that GmLEC2a possibly be capable of to divert the carbon flux more toward lipid biosynthesis rather than protein biosynthesis in soybean. Phylogenetic tree (Supplementary Figure S13) showed that soybean storage proteins are homologs of storage proteins from plants such as Lotus and Medicago. In the light of these results, it can be hypothesized that GmLEC2a has potential to regulate the amino acid metabolism and storage protein synthesis. It can further be anticipated that with advance technology of proteomics these enzymes are either direct or indirect targets of LEC2.

The unique targets of GmLEC2a in lipid biosynthesis, accumulation, and degradation were clearly identified as a result of comparison of LEC2 targets identified in different plant species. The up-regulation of PDH, ACCase, and MAT by both *GmLEC2a* and *AtLEC2* increase the abundance of FA precursors (Kim et al., 2015). The FA condensing enzymes (KASI, II, III), GPDH, LPAT, DGAT, and PDAT were up-regulated by GmLEC2a (Supplementary Table S2) and AtLEC2 (Kim et al., 2015). The transcript of FAD2 and FAD3 were up-regulated in AtLEC2 leaves (Kim et al., 2015), FAD3 transcripts were down-regulated, and FAD7 and FAD8 were up-regulated in transgenic soybean hairy roots (Supplementary Table S2). The regulatory factors (FUS3, LEC1, ABI3, and WRI1) were all over-expressed by GmLEC2a (**Table 1**), AtLEC2 (Kim et al., 2015) and BnLEC2 (Kim et al., 2014). However, Dof11 was only up-regulated by GmLEC2a whereas seed specific TF MYB118 was only found in microarray analysis of AtLEC2 over-expressed leaves. The AtLEC2 over-expressed LACS8 and LACS9 whereas LACS9 in addition to LACS2 was found down-regulated in GmLEC2a transgenic roots while LACS1 transcripts were up-regulated as compared to control (**Table 2**). The GmLEC2a also targets some other transporters such as FAX, TGD, and ABC family transporters that are not yet identified as a target of AtLEC2 (Kim et al., 2015) or BnLEC2 (Kim et al., 2014). The AtLEC2 (Angeles-Núñez and Tiessen, 2010, 2011) and GmLEC2a (**Tables 3, 4** and Supplementary Table S3) were found to be associated with the regulation of protein and starch as well. Identification of LEC2 targets from different crops provides critical information about the carbon partitioning among the storage products.

## Conclusion

As a unique crop serving both as a source of vegetable oil and high quality plant proteins, still very little is known about how soybean seeds synthesize FA, TAG, and protein in coordination with seed development, filling, and maturation. Studies from other plants have demonstrated that transcriptional regulatory network is essential for seed development and sequential biosynthesis and accumulation of various storage substances. The transcriptome data profiling indicates that GmLEC2a can regulate the carbon partitioning for the synthesis of TAGs, carbohydrates, and proteins. Because soybean genome possesses another identical homolog of GmLEC2a and GmLEC2b which shows similar expression patterns, we propose that both GmLEC2s are functional homologs in soybean. The putative targets of GmLEC2a were seed-specifically expressed genes which revealed to be regulated by GmLEC2a in transgenic hairy roots, thus reflecting the functions of GmLEC2 in soybean seed development and seed filling of various storage substances. An overview of storage substances synthesis in soybean seed is shown in Supplementary Figure S14. This framework provides basis for efficient development of customized soybean varieties with desired TAG and protein content to meet the market challenging demand. Although the *in vitro* functions do not always mimic the *in planta* functions, these results shed light on the storage substances precursors partitioning and regulation in soybean seed. This study offers clues about how we can improve a specific component of the soybean seed for broad-spectrum industrial applications.

## Materials and Methods

### Identification of *GmLEC2* in Soybean

The Arabidopsis *LEAFY COTYLEDON 2 (LEC2*) was used as a query to search *Glycine max LEC2* gene by BLASTN analysis. Briefly, the obtained nucleotide sequences were queried against the soybean genome database^1^ in search of homologous sequences. Two homologs of *AtLEC2* were found in soybean Glyma.20G035800.1 (*GmLEC2a*) and Glyma.20G035700.1 (*GmLEC2b*). Moreover, the predicted amino acid sequences obtained from Phytozome were used for multiple sequence alignment (MSA) using Clustal W program available at www.genome.jp/tools/clustalw/. Phylogenetic tree was constructed using Neighbor-Joining method through Mega6. Further, a matrix of pair-wise distance was estimated using a p-distance model. The alignment gaps, missing data, and ambiguous bases were allowed at any position.

### Vector Construction

The open reading frames (ORFs) of *GmLEC2a* (Glyma20G038500.1) was amplified with the cDNA made from soybean developing seeds using pairs of primers mentioned in Supplementary Table S1. Total RNA was extracted from *G. max* developing pods, and 10 μg of total RNA was used to synthesize first-strand cDNA using the first-strand synthesis system (Invitrogen). The *GmLEC2* was amplified and ligated into T-easy vector and sequenced, the cDNA in pDONR221 was recombined into destination vector pB2GW7 by using LR recombinase (Invitrogen).

### Plant Growth Conditions

The soybean (*Glycine max* L.) seeds were germinated in soil in three-gallon pots under photoperiod of 14/10 h with 800 μmol m^-2^.s^-1^ light intensity, 26/20°C day/night temperature, and 60% humidity. Seeds, pods, flowers, leaves, stems, roots, and nodule at different developmental stages were harvested from soybean, grown in a growth chamber under the above mentioned conditions or a natural environment at the fields of Huazhong Agricultural University, Wuhan, China.

*Arabidopsis thaliana* (ecotype Col-0) was used for transformation in this study. The *lec2-1* mutant was taken from Arabidopsis germplasm database (TAIR). The wild-type Col-0, *lec2-1* mutant and the transgenic plants were grown under standard conditions as described previously (Jako, 2001). For consistency in the reproducibility of the oil content measurements, the transgenic lines were always grown with wild-type plants in the same chamber at the same time.

### Soybean Hairy Root Transformation and Analysis of TAG in Hairy Roots

*pB2GW7-GmLEC2a* was transformed by electroporation into *Agrobacterium rhizogene*s strain K599, which was used to transform soybean cotyledons. Seeds of soybean cultivar “Tianlong” was surface sterilized and germinated in sterilized filter papers in petri dishes. The green cotyledons from about 7 days-old germinating soybean seeds were wounded on the surfaces, followed by the infections with *Agrobacterium rhizogene*s K599 bacteria harboring *pB2GW7-GmLEC2a*, or *-GUS* as a control. Generated hairy roots were selected on MS medium containing 7 mg/l phosphinothricin (ppt). The transformed hairy roots expressing *pB2GW7-GmLEC2a* were confirmed with PCR. Then roots were used for further analysis.

### Expression of *GmLEC2a* in Arabidopsis

The binary vector containing the cassette for *35S::GmLEC2a* was transformed into *Agrobacterium tumefaciens* GV3101 by electroporation. Wild-type and transgenic *Arabidopsis thaliana* (ecotype Columbia-0) and mutant *lec2-1* were grown in pot- containing soil in controlled-environment of growth chambers at 22°C with a 16-h light/8-h dark photoperiod. Parental Col-0 and *lec2-1* mutant plants were transformed by using floral dip method. Transgenic plant lines transformed with a vector containing the coding sequence of the *GmLEC2a* were selected based on their resistance to BASTA. Expression of the transgene in developing seeds was confirmed by RT-PCR. The dry seeds of T3 Arabidopsis transformants were analyzed for oil contents and FA composition.

### Quantification of TAG and Analysis of FA Composition

Total lipid extraction and TAG content and composition determination were done according to previously described methods with slight modifications (Browse et al., 1986). Briefly, total lipids from soybean hairy roots (∼ 0.2 g fresh tissues) were extracted with 4 ml of 4 M HCl in glass tubes tightly with Teflon-lined caps at room temperature for 30 min, then in a 100°C water bath for 10 min. After cool tubes were centrifuged hairy root powder was extracted with 4 ml of hexane: isopropanol (3:2, v/v). The upper hexane layer of the extractions was removed into a new glass tube and evaporated under a slow stream of N_2_ gas. The residues were dissolved in 50 μl hexane for TLC analysis. The TAG from soybean hairy roots was resolved by TLC on a silica plate (SIL GF254, 0.25 mm). The plate was developed with hexane/diethyl ether/acetic acid (80:20:1, v/v/v), essentially according to the method as previously described (Chen et al., 2016). Fatty acid methyl esters (FAMEs) were prepared by heating the dry TAG materials at 85°C for 30 min in 1 M HCl in dry methanol. FAMEs then were dried under nitrogen gas and resuspended in 200 μl of hexane for GC analysis. The TAG content and composition from Arabidopsis seeds were measured according to a previously reported method (Chen et al., 2016). Briefly, approximately 10 mg of seeds were weighed in a 13 × 100 mm glass tube with a teflon layered screw-cap. Thereafter, 1.5 ml of 2.5% sulphuric acid in methanol, 400 μl toluene, and 100 μl of 1 mg/ml triheptadecanoin in toluene (Nu-Chek Prep, Elysian, MN. United States) as an internal standard were added to each sample tube. All sample tubes were heated at 90°C for 1 h. The FAMEs generated by above *trans*-esterification reaction were extracted by addition of 1 ml hexane and 1.8 ml H_2_O. After thorough mixing and centrifugation hexane layer was recovered and analyzed with GC. FA content and composition on TAGs from seeds or purified with TLC from total lipids of hairy roots were analyzed with an Agilent 7890A GC system with flame ionization detector (FID). Oil content was calculated by FID response of sample components relative to 17:0 methyl ester from the internal standard triheptadecanoin.

### Quantitative RT-PCR (qRT-PCR) Analysis of Gene Expression

The total RNAs from tissues of soybean plants and Arabidopsis leaves were isolated following the protocol provided with RNA isolation kit supplied by Biotech, Beijing, China. Briefly, 10 μg of total RNA was treated with RNase-free DNaseI (Promega, Madison, WI, United States) to remove any genomic DNA contamination for each sample. First-strand cDNA was synthesized from 2 μg total RNA using the MMLV first strand synthesis kit (Invitrogen^TM^). Each cDNA sample was 20-fold diluted in sterile water for qRT-PCR reaction. The expression was normalized using soybean *ACTIN* as internal control. qRT-PCR reactions were performed in 96-well plates (iQ5 Real Time PCR System; Bio-Rad) for all tissues tested, and data were analyzed according to methods described previously (Chen et al., 2016).

### Histochemical Staining for Starch

The leaves of 4-week-old Arabidopsis plants were subjected for starch-iodine staining. The chlorophyll was removed by boiling leaves in methanol at 85°C for 5 min in a water bath. After chlorophyll extraction, the leaves were incubated in iodine solution for 10 min. The leaves, which contained iodine-stained starch, were photographed with a digital camera.

### Spectrophotometric Determination of Starch

Starch in leaf samples was estimated by using the method described previously (Séne et al., 1997) was used. A sample (0.05 g seeds) was twice shaken in 70% (v/v) aqueous acetone (2 ml) to eliminate lipids and then centrifuged at 1600 *g* for 15 min. The starch in the dry pellet was suspended in H_2_O (1 ml), solubilised in 5 M NaOH (4 ml) with constant stirring for 1 h at room temperature, then neutralized with HCL (0.25 ml). Add 1 ml of iodine solution (4 g potassium iodide + 1.27 g iodine/100 ml H_2_O) in the tubes. Color was allowed to develop for 10 min and absorbance was read at 660 nm using MAPADA spectrophotometer.

### Protein Extraction

Total protein was extracted from 0.2 g of each sample of transgenic and control hairy roots. Roots were grounded into fine powder in liquid nitrogen. 1 ml of extraction buffer (50 mM Tris HCl, 80mM KCl and 2mM EDTA, pH 7.5), 50 μl PMSF (1 M) and 50 μl DDT (1 M) was added following sonication for 1 h. The total protein from Arabidopsis seeds was extracted using method previously reported (Damania et al., 1983). Absorbance was recorded at 595nm with comasine blue G250, BSA is used as an internal standard.

### cDNA Library Construction for Illumina Deep Sequencing

Total RNA was extracted with Trizol reagent (Invitrogen, Waltham, CA, United States) or RNA kit (Biotech, Beijing) following the manufacturer’s instructions. RNA integrity was confirmed by using the 2100 Bioanalyzer. A total of 0.5–2 μg RNA per sample was used for cDNA library preparation using the TruSeq RNA sample preparation kit (Illumina, San Diego, CA, USA). Each library was sequenced on an Illumina HiSeq2500 instrument. Approximately 70 million 100 bp pair-end reads were generated for each sample. For processing of data from RNA-Seq experiments, the raw data were first processed using the NGS QC Toolkit, and the clean data were obtained by removing reads containing adapter, poly-N and low-quality reads. The analyses on clean data with high quality control, differential gene and transcript expression, total reads mapped to the soybean genome in RNA-Seq experiments were conducted by the Biotech Company Novogene Corporation. The fragments per kilobase of transcript per million mapped reads (FPKM) and transcript level per million count values were calculated using eXpress. DGE was analyzed by using the DESeq (2012) R package. Hierarchical cluster analysis based on the differentially expressed genes (DEGs) were filtered with log2 fold change (Log_2_ FC) > 1 or < –1 in each pairwise comparison.

## Statistic Analysis

Most experimental data were obtained from at least three independent experiments and were analyzed using Student’s *t*-test. The significant differences between two tails of data represent 95% confidence limits. Representative of photos or images were shown from at least three experimental repeats.

## Author Contributions

JZ planned and designed the research. SM, MA, GZ, BC, BH, and JY performed experiments and analyzed data. SM and GZ conducted bioinformatics analyses. JZ and SM wrote the manuscript.

## Conflict of Interest Statement

The authors declare that the research was conducted in the absence of any commercial or financial relationships that could be construed as a potential conflict of interest.

## Footnotes

1 https://phytozome.jgi.doe.gov/pz/portal.html
