# Supplementary material for: Soybean LEC2 Regulates Subsets of Genes Involved in Controlling the Biosynthesis and Catabolism of Seed Storage Substances and Seed Development
Source: Front Plant Sci. 2017 Sep 20;8:1604. doi: 10.3389/fpls.2017.01604 (PMC5611487; doi:10.3389/fpls.2017.01604)
Supplement: Supplementary file 1 [file Data_Sheet_1.doc]

**Supplementary data information for**

**Soybean LEC2 regulates subsets of genes involved in controlling the biosynthesis and catabolism of seed storage substances and seed development**

Sehrish Manan^1^, Muhammad Zulfiqar Ahmad^1,2^, Gaoyang Zhang^1,2^, Beibei Chen^1^, Basir Ul Haq^1^, Jihong Yang^2^,.Jian Zhao^1, 2^*

^1^National Key Laboratory of Crop Genetic Improvement, Huazhong Agricultural University, Wuhan 430075, China. ^2^ State Key Lab of Tea Plant Biology and Utilization, College of Tea and Food Science and Technology, Anhui Agricultural University, Hefei 230036, China

**Supplementary contents:**

**Supplementary Figure S1. *GmLEC2a* expression in public database (phytozome)**

**Supplementary Figure S2. Expression of GmLEC2a in representative lines of Arabidopsis lec2 mutant.**

**Supplementary Figure S3. Expression of GmLEC2a in representative lines of wild-type (Col-0) Arabidopsis**

**Supplementary Figure S4. Expression of GmLEC2a in representative transgenic hairy roots (HR).**

**Supplementary Figure S5. *GmLEC2a* ectopic expression alters the total protein content**

**Supplementary Figure S6. The RY elements in the promoter region of *GmLEC2a* target genes.**

**Supplementary Figure S7. Phylogenetic analysis of Transcription factors of seed development in *G.max* and other crops.**

**Supplementary Figure S8. Maximum likelihood phylogeny of FA and TAG biosynthesis genes in *G.max* and other plants.**

**Supplementary Figure S6. Maximum likelihood phylogeny of lipid transporters in *G.max* and other plants.**

**Supplementary Figure S10. Phylogenetic relationship between ABC family members in soybean.**

**Supplementary Figure S11. Phylogenetic relationship between LACS genes in *G.max*.**

**Supplementary Figure S12. Maximum likelihood phylogeny of sucrose synthases and sucrose transporter in *G.max* and other plants.**

**Supplementary Figure S13. Phylogenetic analysis of genes encoding enzymes of TCA cycle and protein metabolism in G.max and other plants.**

**Supplementary Figure S14. Overview of metabolic routes leading to major storage compounds in soybean seed.**

**Supplementary Table S1. List of Primers used in this study**

**Supplementary Table S2. List of enzyme encoding genes in *de novo* FA and TAG biosynthesis**

**Supplementary Table S3 . List of genes encoding enzymes of TCA cycle**

**
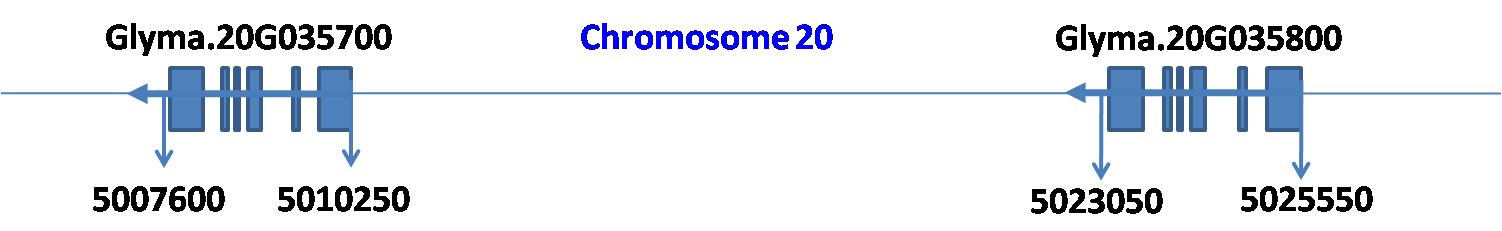
**

**
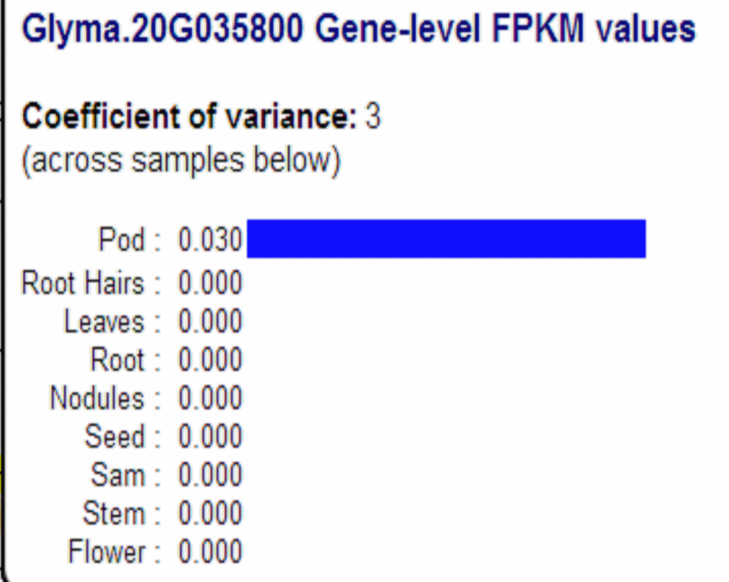

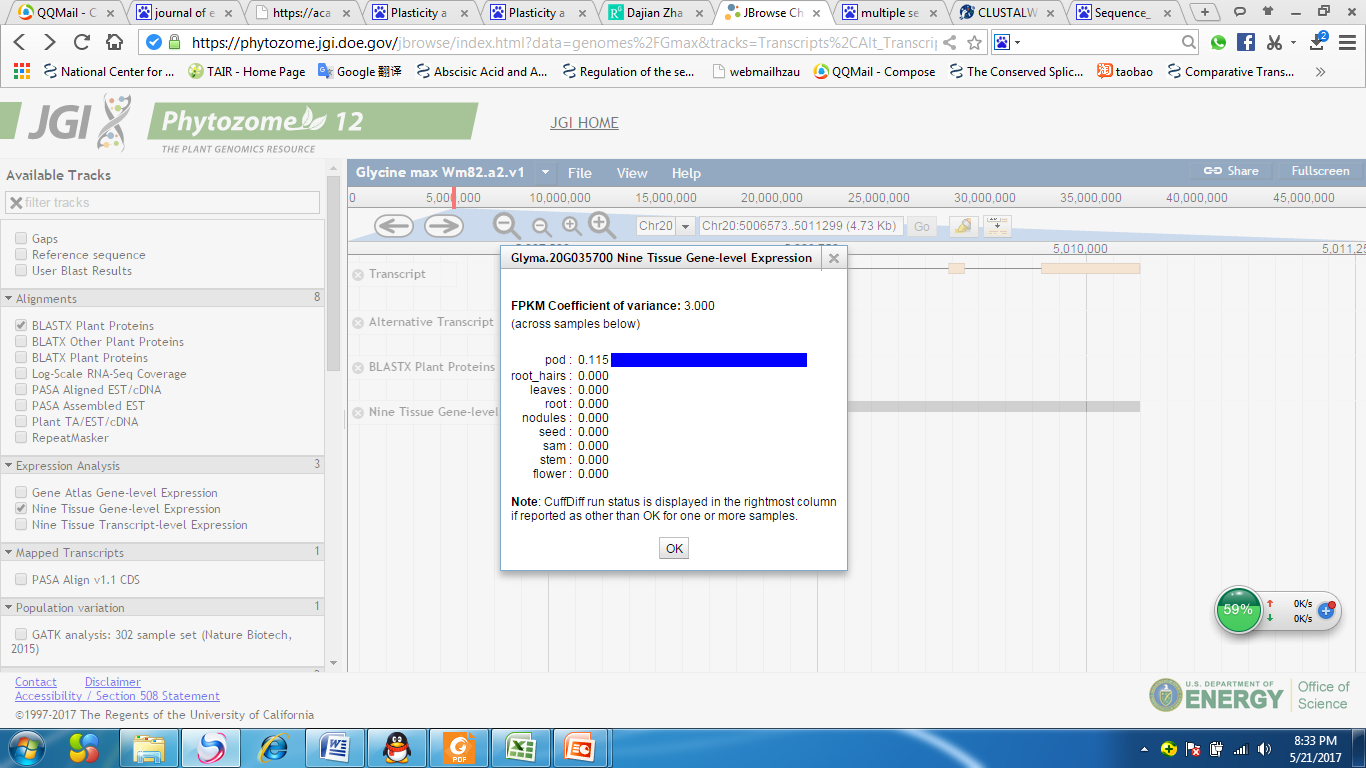
**

**Supplementary Figure S1. *GmLEC2a* and *GmLEC2b* gene structures and expression as shown in public database (phytozome:** [**https://phytozome.jgi.doe.gov/jbrowse/**](https://phytozome.jgi.doe.gov/jbrowse/)**)**


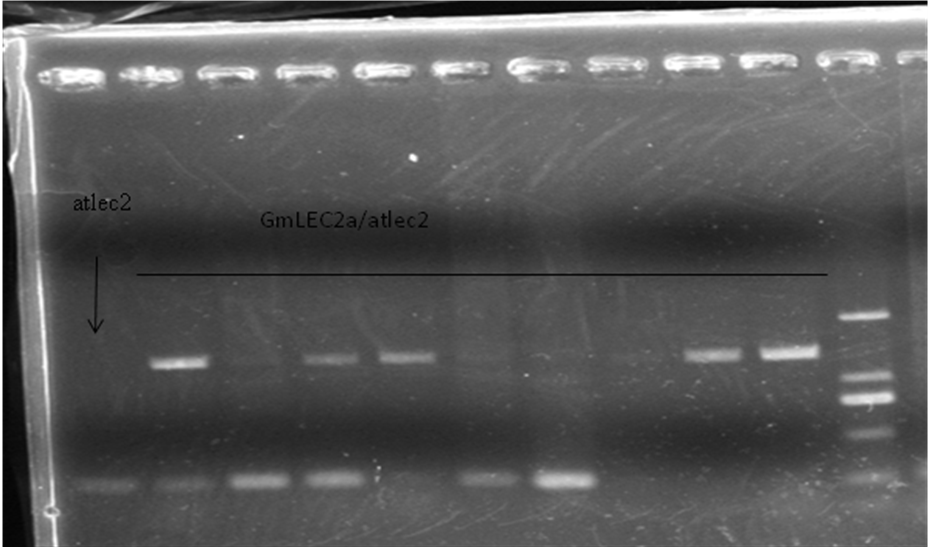


**Supplementary Figure S2. Expression of GmLEC2a (1134bp) in representative lines of Arabidopsis lec2 mutant.**


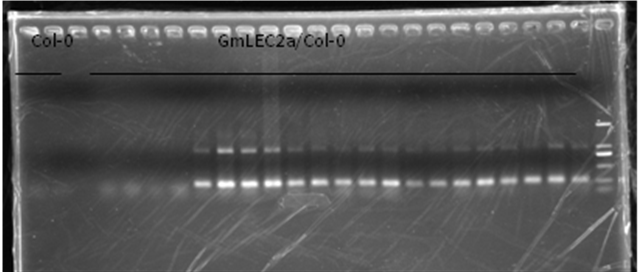


**Supplementary Figure S3. Expression of GmLEC2a (1134bp) in representative lines of wild-type (Col-0) Arabidopsis.**


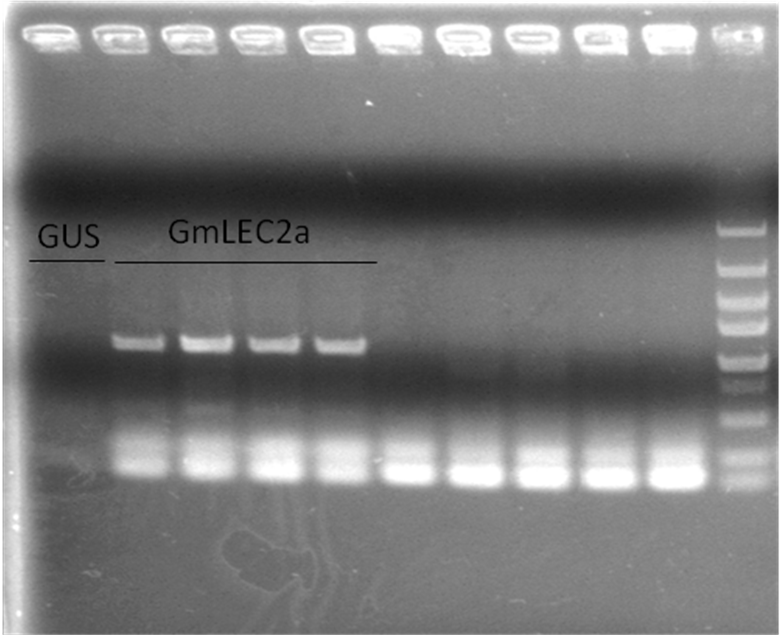


**Supplementary Figure S4. Expression of GmLEC2a in representative transgenic hairy roots (HR). *GUS* gene is used as a control.**

**
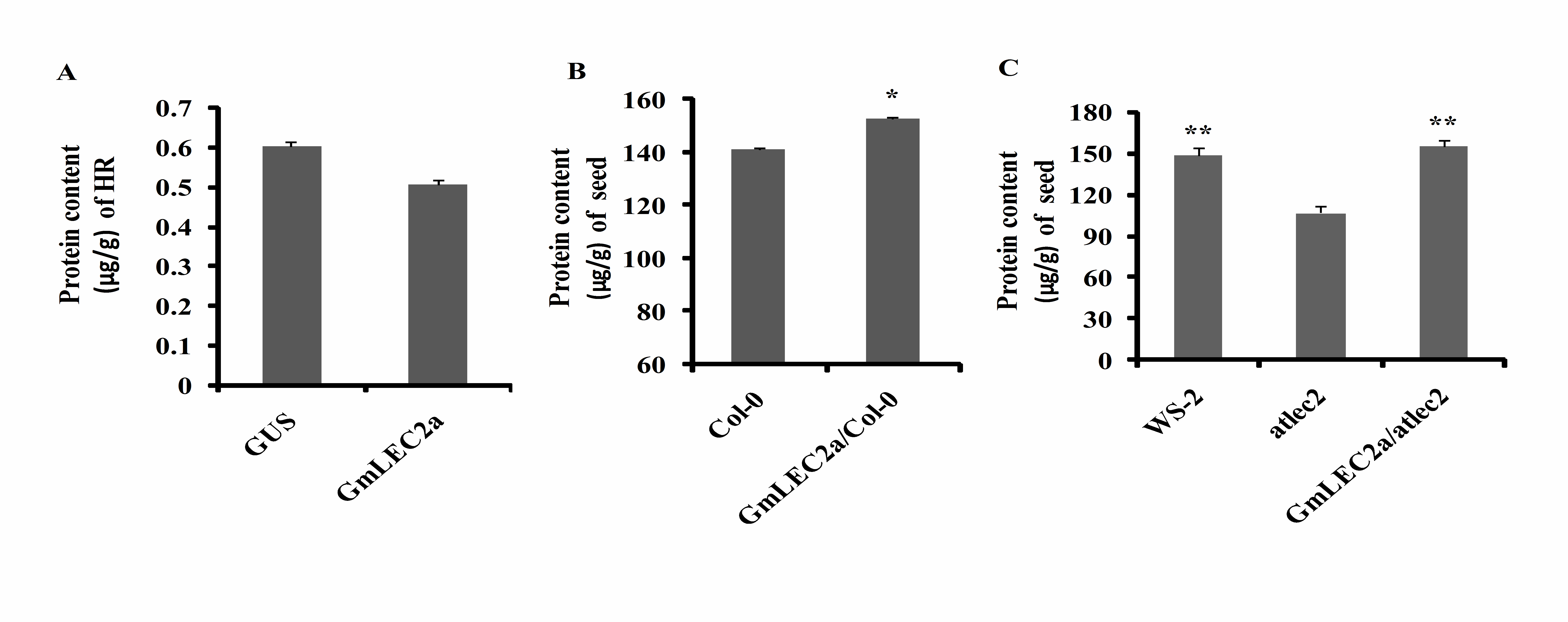
**

**Supplementary Figure S5. GmLEC2a ectopic expression alters the total protein content. A.** The total protein in GmLEC2a over-expressed hairy roots (HR) was reduced compared to control hairy roots (*GUS).* **B.** The total protein level in over-expressed GmLEC2a Arabidopsis seeds was higher relative to wild-type (Col-0) seeds. **C.** Protein content of *atlec2* mutant seeds was lower than the wild-type (WS-2) and *GmLEC2a/atlec2* expressed mutant seeds. All data are three biological replicates and are expressed as means ± SD. **, P< 0.01 and *, P < 0.05 by Student’s *t*-test (n = 3).

**
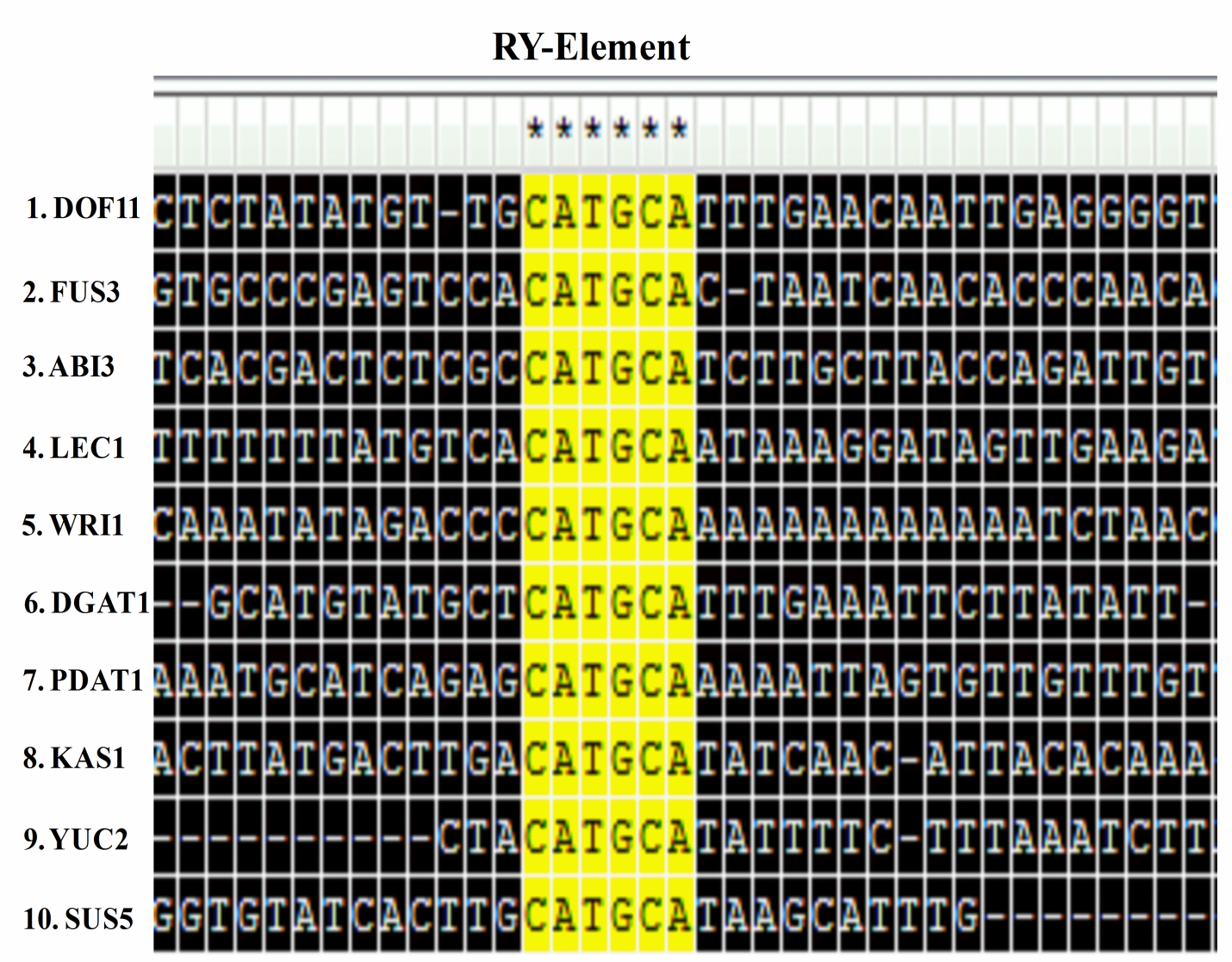
**

**Supplementary Figure S6. The RY elements in the promoter region of *GmLEC2* target genes.** The yellow highlighted part shows the RY element in the upstream region of *GmLEC2* targeted genes in soybean.

**
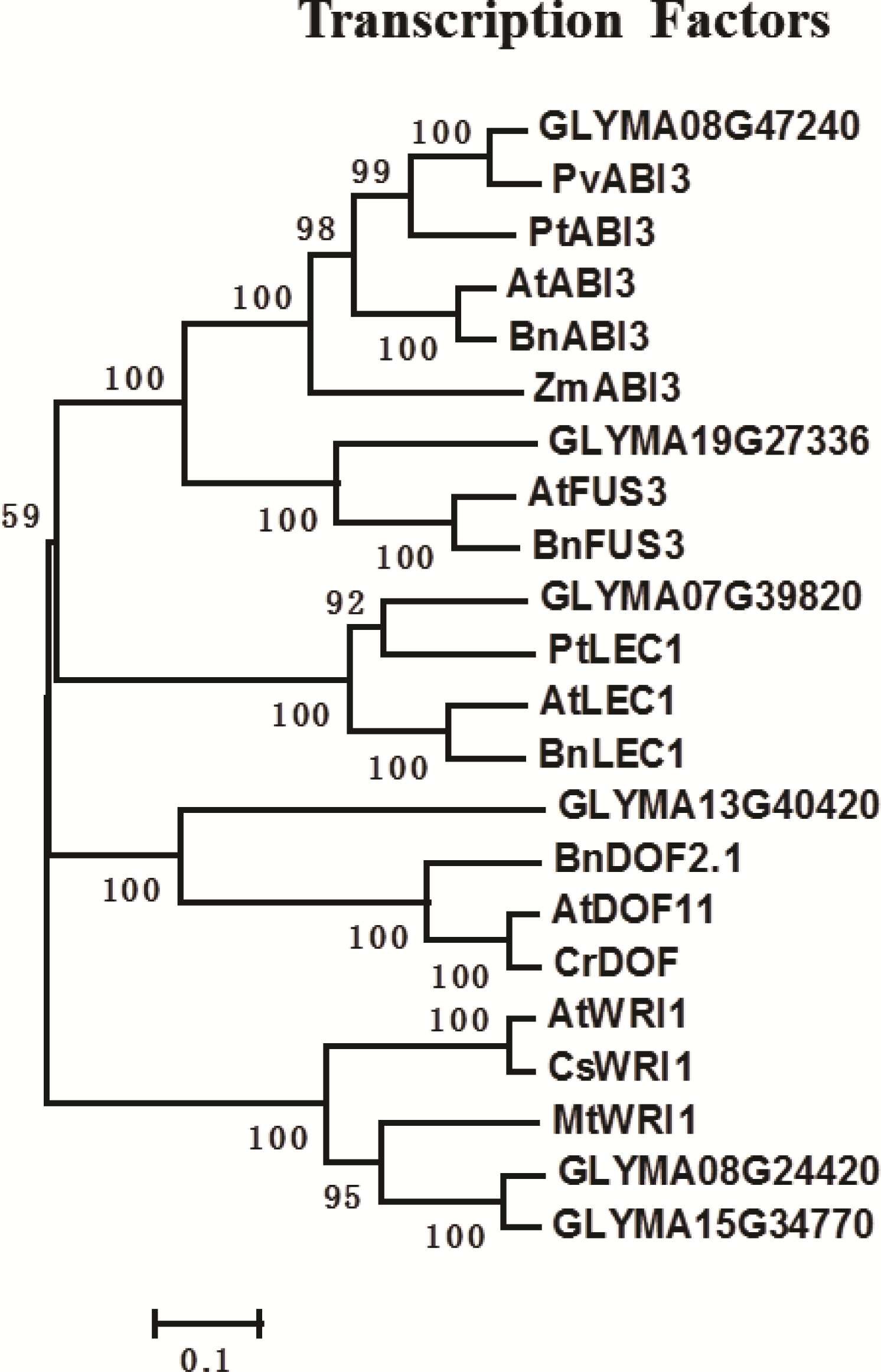
**

**Supplementary Figure S7. Phylogenetic analysis of Transcription factors of seed development in *G.max* and other crops.** ABI3, Abscisic acid insensitive 3; FUS3, FUSCA3;, LEC1, Leafy cotyledon 1; WRI1, Wrinkled 1; Dof, DNA binding with one finger**.**

**
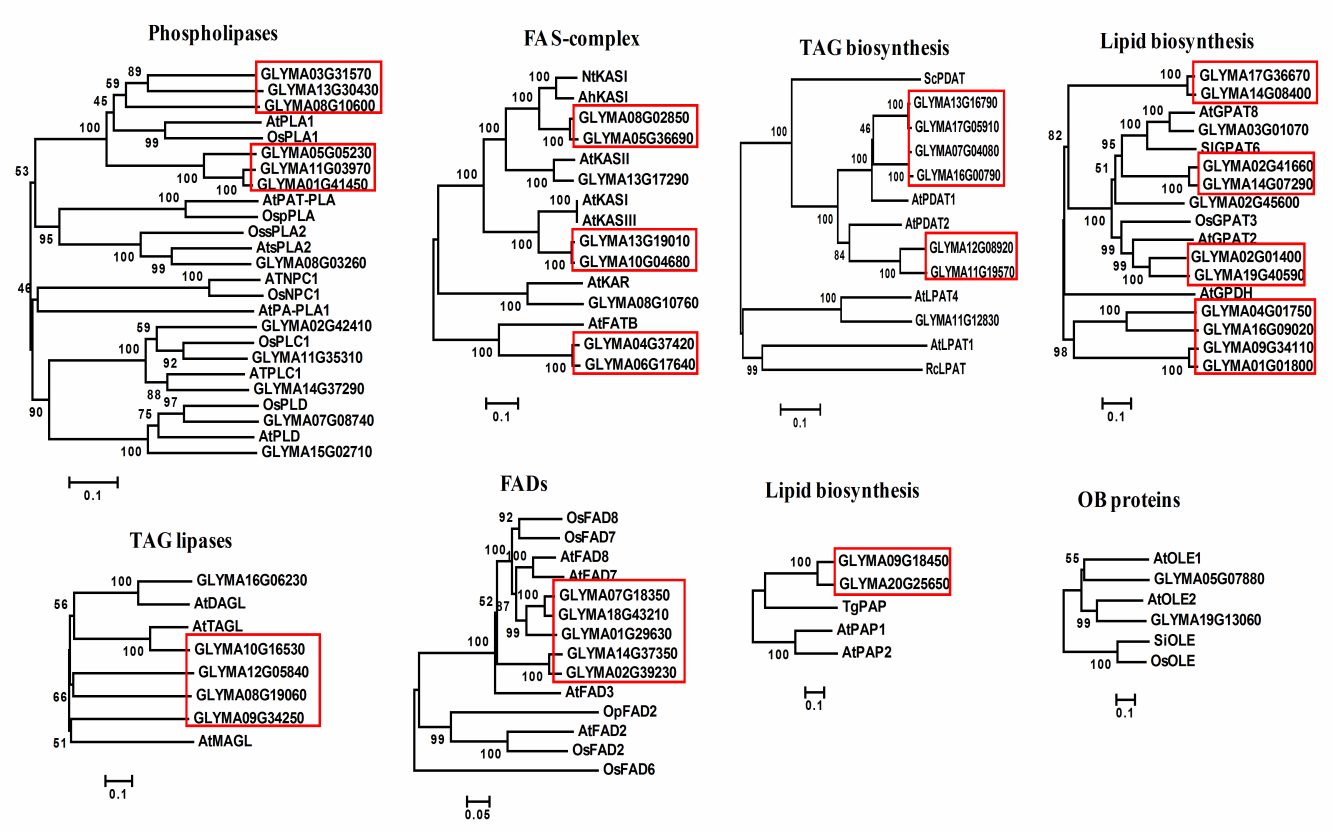
**

**Supplementary Figure S8. Maximum likelihood phylogeny of FA and TAG biosynthesis genes in G.max and other plants.** PLA/B/C, Phospholipase A/B/C; KAS**,** 3-oxoacyl-[acyl-carrier-protein] synthase; KAR, 3-oxoacyl-[acyl-carrier-protein] reductase; FATB Fatty acyl thioesterase B; PDAT, Phospholipid:diacylglycerol acyltransferase; LPAT, Lysophosphatidyl acyltransferase; PAP, Phosphatidic acid phosphatase; GPAT, Glycerol-3-phosphate acyltransferase, GPDH, Glyceraldehyde-3-phosphate dehydrogenase, TAGL, TAG lipase; DAGL; DAG lipase; FAD, Fatty acid desaturases; OB oil bodies; OLE; Oleosin.

**
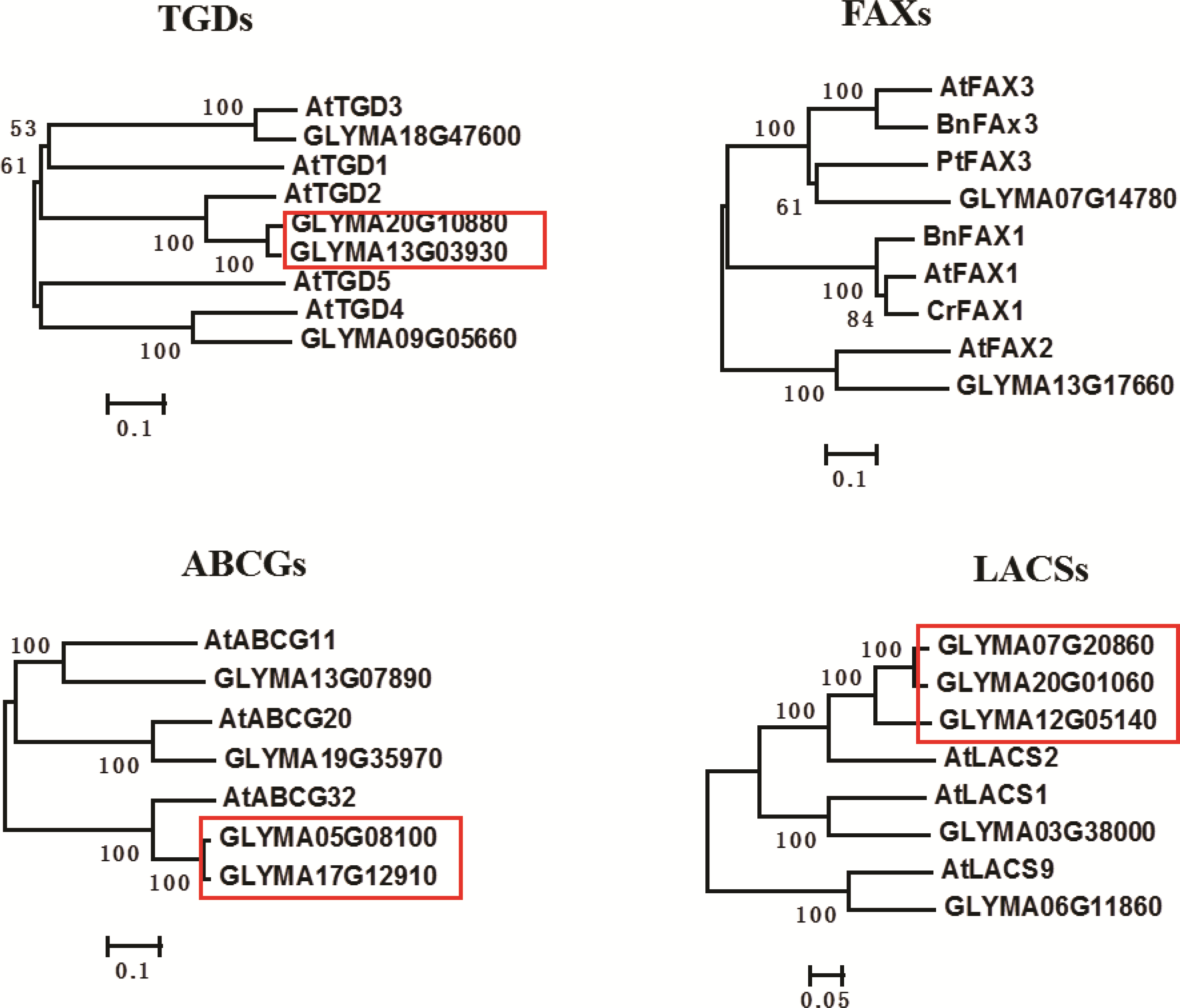
**

**Supplementary Figure S9. Maximum likelihood phylogeny of lipid transporters in *G.max* and other plants.** TGD Trigalactosyldiacylglycerol; FAX, Fatty acid export; ABCG, ABC transporter family Gmember; LACS, Long chain acyl Co-Asynthase.


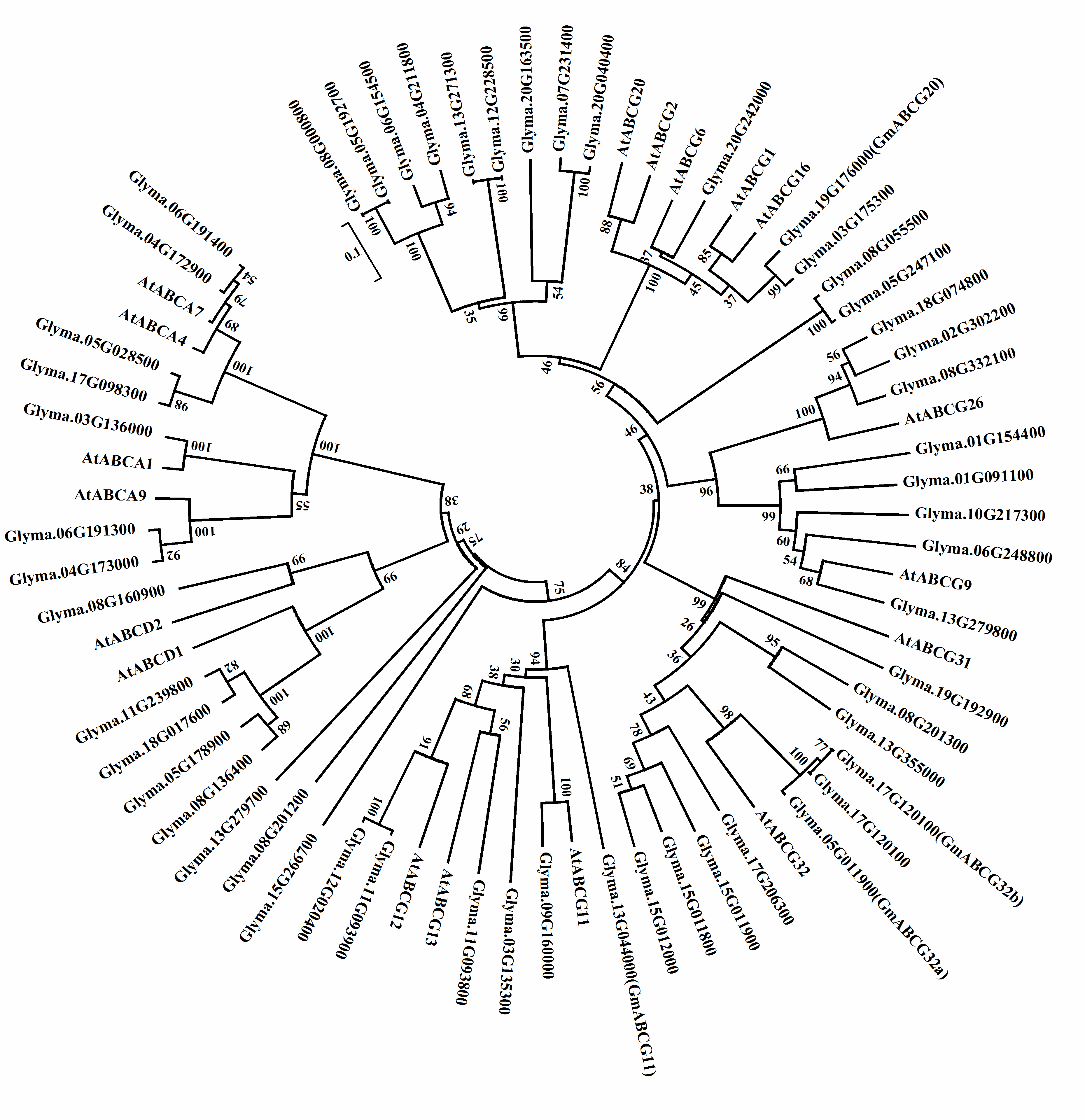


**Supplementary Figure S10. Phylogenetic relationship between ABC family members in soybean.**


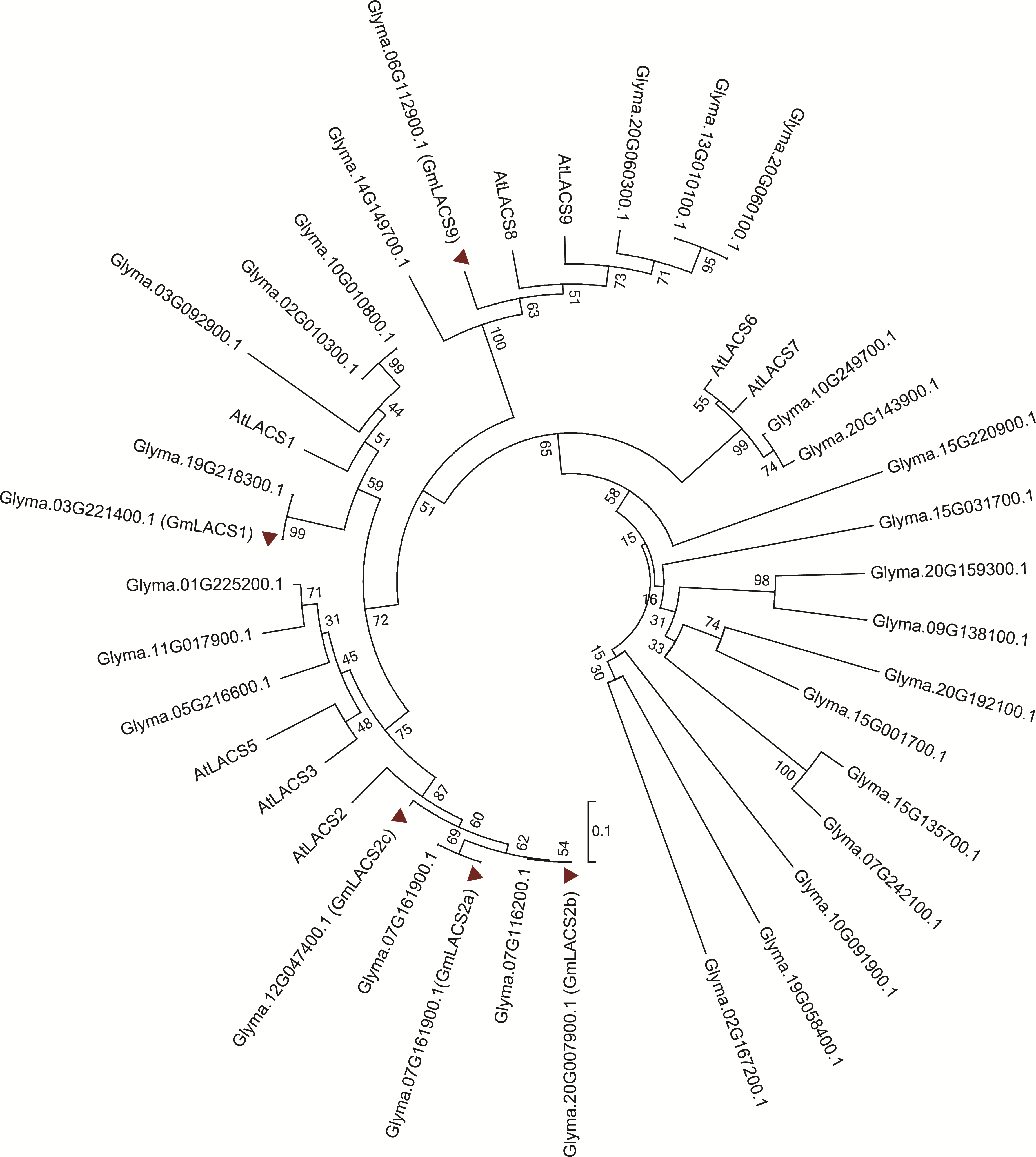


**Supplementary Figure S11. Phylogenetic relationship between LACS genes in *G.max*.**

**
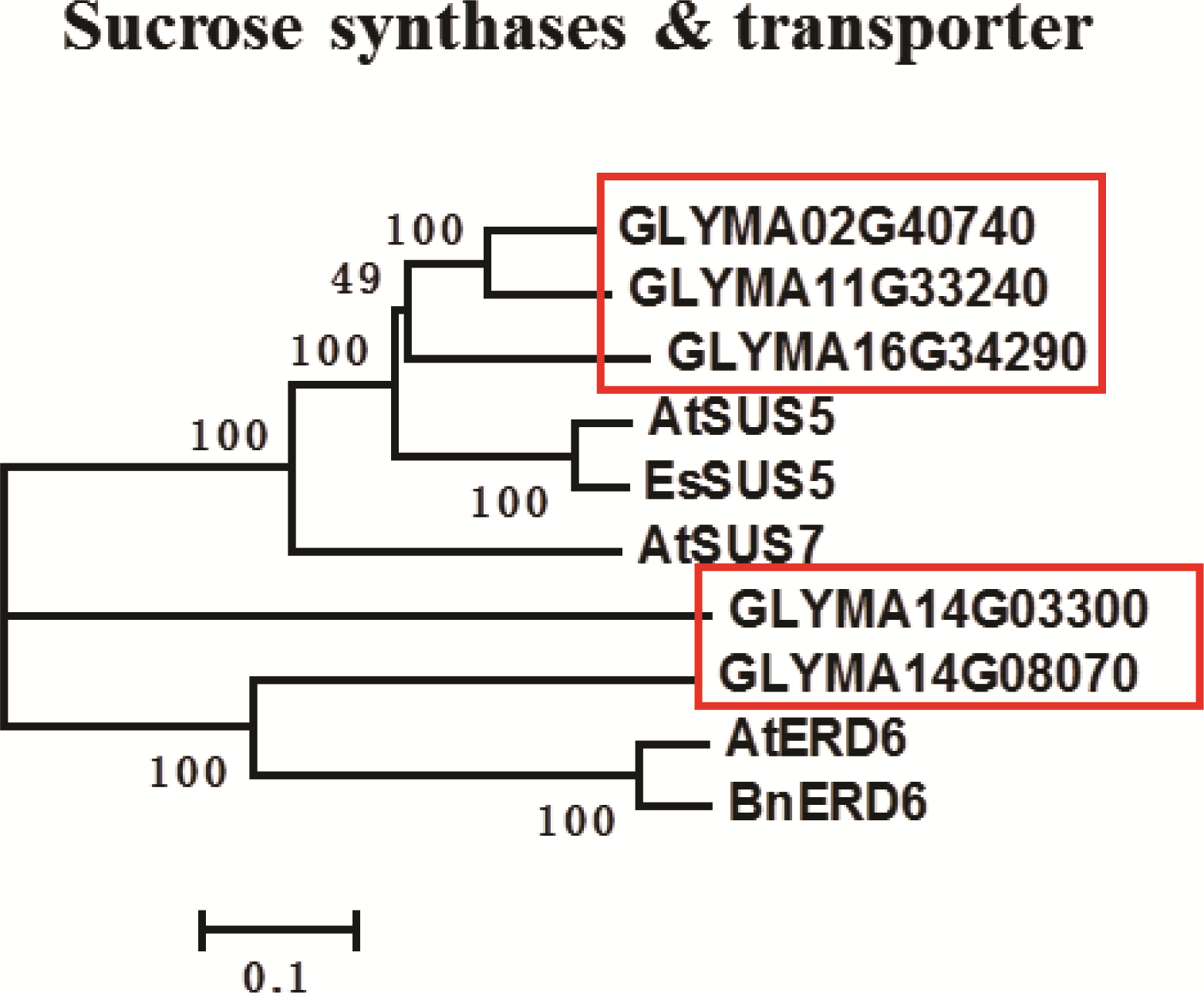
**

**Supplementary Figure S12. Maximum likelihood phylogeny of sucrose synthases and sucrose transporter in *G.max* and other plants.** SUS, Sucrose synthase, ERD6, Early response to dehydration 6.

**
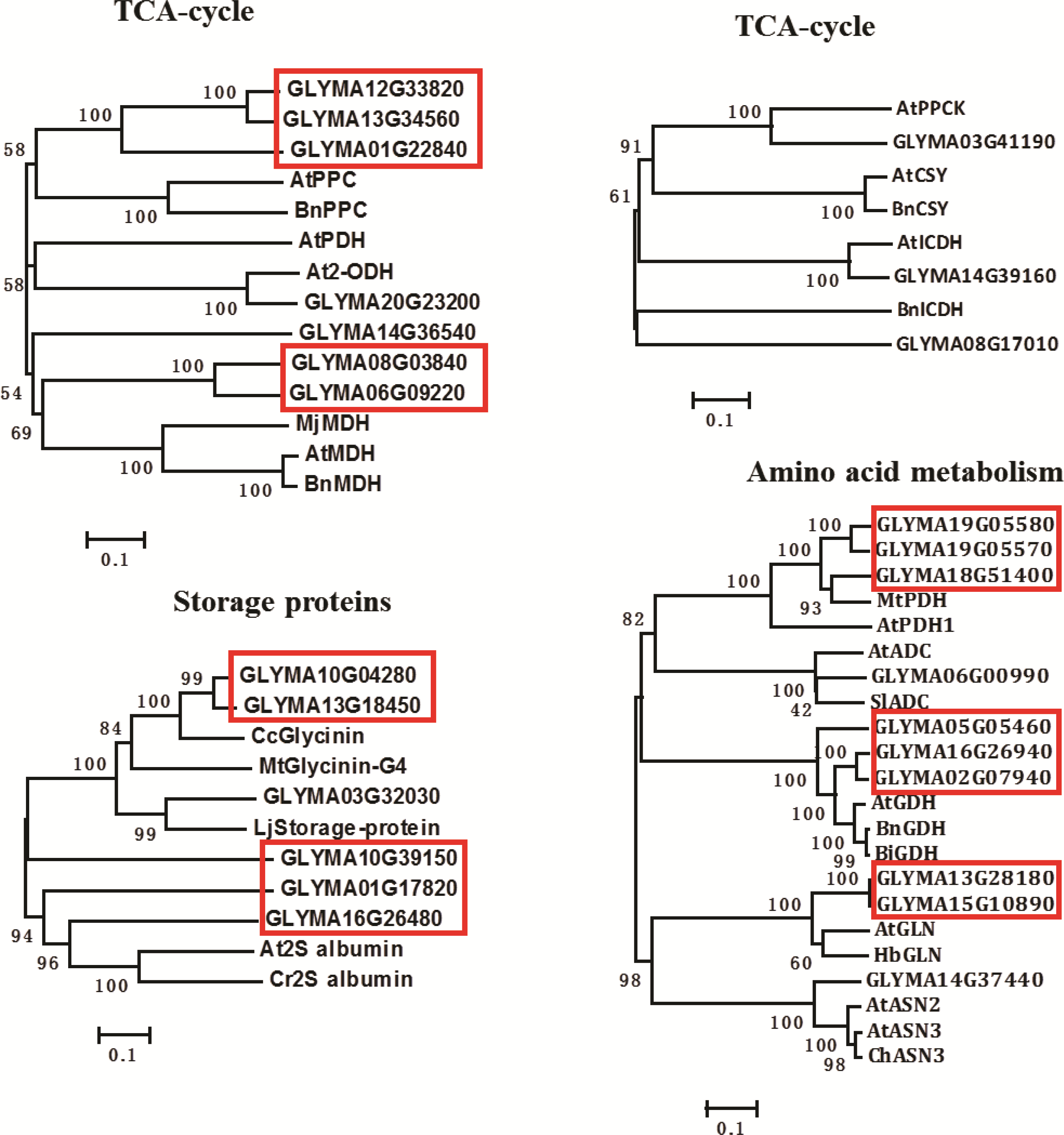
**

**Supplementary Figure S13. Phylogenetic analysis of genes encoding enzymes of TCA cycle and protein metabolism in *G.max* and other plants.** PPC, Pyruvate carboxylase; PPCK, Pyruvate carboxylase kinase; ODH, oxoglutarate dehrdogenase ; PDH, Pyruvate dehrdogenase; MDH, Malate dehrdogenase; CSY, citrate synthase; ICDH, Iso citrate dehydrogenase; ADC, Arginine decarboxylase; GDH, Glutamate dehydrogenase; GDN, Glutamine Synthase; ASN, Asparagine synthase

**
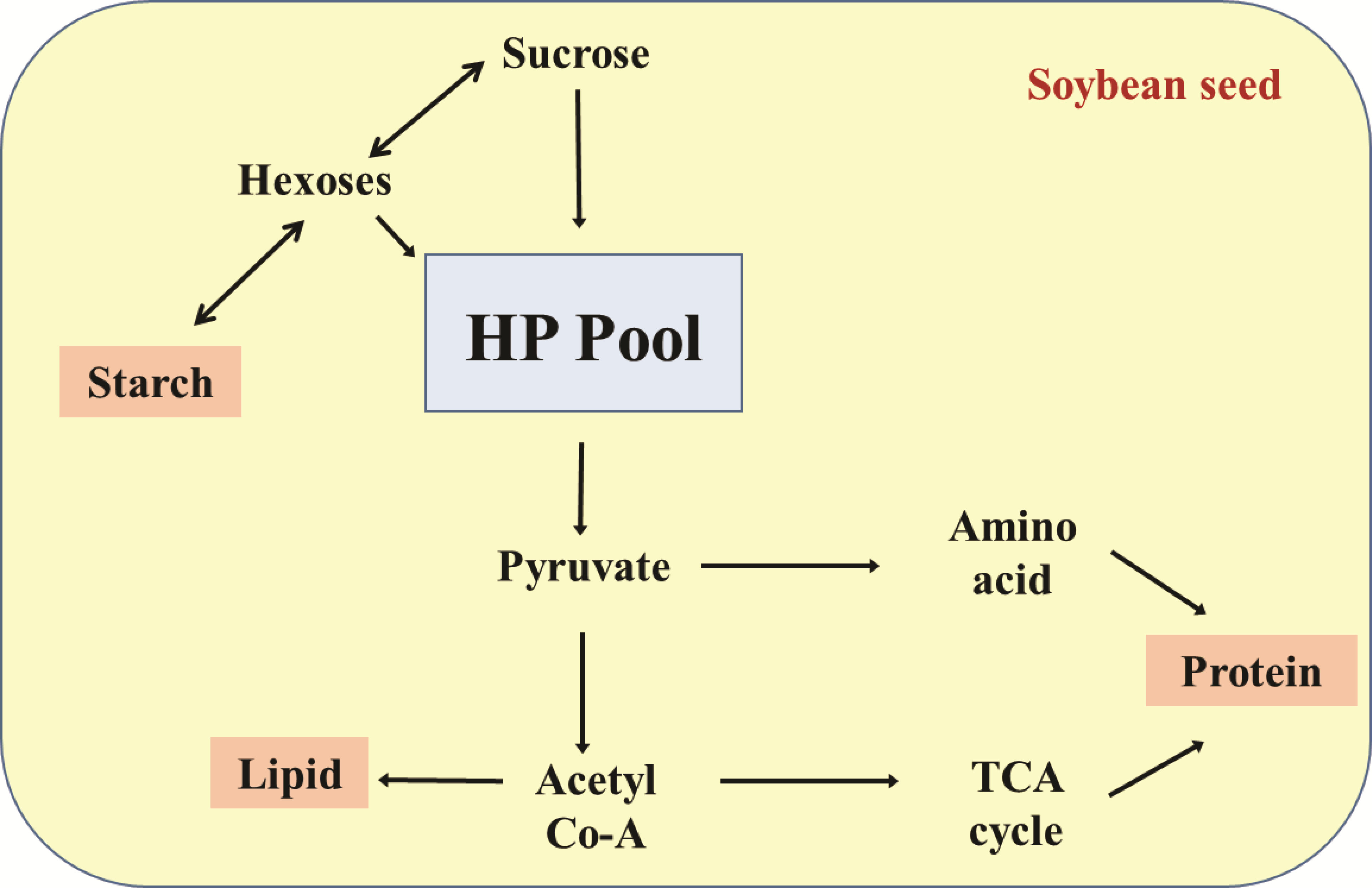
**

**Supplementary Figure S14.** **Overview of metabolic routes leading to major storage compounds in soybean seed**. Sucrose act as a major carbon source for the synthesis of all storage compounds. Sucrose and hexoses enter to hexose-P pool (HP-Pool) that further provide precursor for different storage compounds synthesis. It is clear from this scheme that several reactions/enzymes/pathways coordinated in order to preferentially partition carbon to starch, oil or proteins.

**Supplementary** Table S1. List of Primers used in this study

| Primer name | Primer sequence 5’ - 3’ | Used for |
| --- | --- | --- |
| GmLEC2-F | GCATGGAAAACTTTTTTGTGCCAT | For cloning cDNA into T-easy vector |
| GmLEC2-R | GCCGCGCTACCAAATTTGATAAGTA |
| GmLEC2-LF | GGGGACAAGTTTgtacaaaaaagcaggctTCATGGA  AAACTTTTTTGTGC | For cloning into pDONOR |
| GmLEC2-LR | GGGGACCACTTTGTACAAGaaagctgggtACTACC  AAATTTGATAAGTA |
| GmLEC2-qRT-F | GGTGGCAATGGCTCATCTTCATCAA | Used for qRT |
| GmLEC2-qRT-R | GGAAACATAGGGAAAGGTTGTCCTG |
| GmACTIN-F | CTTCCCTCAGCACCTTCCAA |
| GmACTIN-R | GGTCCAGCTTTCACACTCCAT |
| AtACTIN-F | TGGATATCAGGAAGGATCTG |
| AtACTIN-R | GTGCAACGACCTTAATCTTC |
| TGD3-F: | ATGCTTCTACTAGAATCCCTCC |
| TGD3-R: | TGAATGGCGGAAGAGTCCT |
| TGD4-F: | ATGATAGAGTGCCACCACCTA |
| TGD4-R: | GCTGACACGTGAGGATTCGA |
| FAX3-F: | ATCTTCGTCGGAATCCCCACTC |
| FAX3-R: | GTGTCCAGAGCTTGCTTCCAC |
| Sdap1-F: | ACGAGGCTAGTGTTGACCCTT |
| Sdap1-R: | ATGCAACCATGACACAATGGGTC |
| PDAT1-F: | AGAGATGGTCGTGCGTGGATA |
| PDAT1-R: | CAAGCCCTCCTTCCTGAGC |
| AGL15-F: | ATGGGTCGAGGGAAAATCGAGA |
| AGL15-R: | GAGAAAACTATGACAGCAACCTCG |
| GPAT4-F: | GTAGGAAGACAGTGTCATTCA |
| GPAT4-R: | CCTCAGTTCTGTGATTAGCAATA |
| Ole1-F: | ATGGCGGACCAATACCCAAGC |
| Ole1-R: | GGTTAGGCCCGTCAGGAGG |
| GmDGAT1a-F | ACTCCATCAGCAGCGACGC |
| GmDGAT1a-R | GTGACTCTGCAACTGACGGA |
| GmDGAT2D-F | AGAGCATGGCGGCGGAAC |
| GmDGAT2D-R | GACGAATCACCAAATTCTTC |
| GmWRI1a-F | GACGAATCACCAAATTCTTC |
| GmWRI1a-R | TTGTTGTAGATGATGGTGAAGA |
| GmWRI1b-F | AAGCATGATGCATGTGGATA |
| GmWRI1b-R | TAGTTGTAGATGATGGTGAAGA |
| GmABI3-F | GGGGGGTGGTGATAATTGCACC |
| GmABI3-R | AAGGCAGACCGGTCCACAGCTG |
| GmFUS3-F | CGTTCCAGGGAACAACACCAACA |
| GmFUS3-R | GAGAACGAGGCTTGTTGTGCTT |
| GmLEC1-F | ATGGAAACTGGAGGCTTTCACGG |
| GmLEC1-R | GTAGTCTAGTGTCACGTCGGTGT |
| GmABCG11-F | TGTAATGCCACTAACTACGAAC |  |
| GmABCG11-R | TGCAAATCCTCCCATGTTAG |  |
| GmABCG20- F | CTGTCCACGAAGACAGGCTTC |  |
| GmABCG20-R | TCGAGAACATGGTGGGGTGGTG |  |
| GmABCG32-F | ATCGCCGTCTTTCAGGGAG |  |
| GmABCG32-R | CTGACGTCGATCTCTTTCATGT |  |
| GmLACS2-F | ACAATATGTTGGGTAGGCGTC |  |
| GmLACS2-R | GATCCATTCAGGGCAGTTGGA |  |
| GmERD6-F | TGAGATATCACCTCCAAACTTGAG |  |
| GmERD6-R | GGCCAGGTATCAATATTGTACA |  |
| GmLACS1-F | AGAGTTTTGCTGCTAAGGTTGAAG |  |
| GmLACS1-R | CACGCCATCCCAACATACGGTT |  |
| GmPXA1-F | AGTCACTCCAAGTTCTTGCTGC |  |
| GmPXA1-R | CAGCACGAAATAGGAAGCC |  |
| GmBCCP1-F | ATGGCATCCTCGTTGGCACCA |  |
| GmBCCP1-R | GGAACTATCAAGGGCAACCTCATT |  |
| GmGL2-F | ATGGCCGCCGACATGTCCAA |  |
| GmGL2-R | TTCACCCTCCTCCACCGAA |  |
| GmFAE1-F | ATGTCCACGTCACCGTCCG |  |
| GmFAE1-R | GAAACGACGACGTAAGAGGCGAT |  |
| GmSUS5-F | CTGGCGATGATCAAGCAATAGTGG |  |
| GmSUS5-R | CTCAGAGACATTAACACGGACAT |  |

**Supplementary Table S2. List of enzyme encoding genes in *de novo* FA and TAG biosynthesis**

| **Symbol** | **Enzyme name** | **Glyma ID** | **Log2 FC** | **Up/Down** |
| --- | --- | --- | --- | --- |
| ACCase | Acetyl-CoA carboxylase | GLYMA11G35740 | 1.1025 | Up |
|  |  | GLYMA18G02670 | 1.5621 |  |
| KASI | 3-oxoacyl-[acyl-carrier-protein] synthase I, | GLYMA13G19010 | 1.444772 | Up |
|  |  | GLYMA08G02850 | -1.45622 | Down |
|  |  | GLYMA05G36690 | -1.96923 |  |
| KASII | 3-oxoacyl-[acyl-carrier-protein] synthase | GLYMA13G17290 | 1.11952 | Up |
| KASIII | 3-oxoacyl-[acyl-carrier-protein] synthase III | GLYMA10G04680 | 1.488158 | Up |
| KAR | 3-oxoacyl-[acyl-carrier-protein] reductase | GLYMA08G10760 | 1.168271 | Up |
| FATB | Palmitoyl/stearoyl-acyl carrier protein thioesterase | GLYMA04G37420 | -1.3318 | Down |
|  |  | GLYMA06G17640 | -1.12124 |  |
| GPDH | glyceraldehyde-3-phosphate dehydrogenase | GLYMA04G01750 | -2.00358 | Down |
|  |  | GLYMA16G09020 | 1.175588 | Up |
| GPAT | Glycerol-3-phosphate acyltransferase | GLYMA14G08400 | -1.18249 | Down |
|  |  | GLYMA03G01070 | -1.26783 |  |
|  |  | GLYMA02G45600 | -1.41669 |  |
|  |  | GLYMA02G41660 | -1.53217 |  |
|  |  | GLYMA14G07290 | -2.66361 |  |
|  |  | GLYMA17G36670 | 0.953107 | Up |
|  |  | GLYMA02G01400 | 4.24021 |  |
|  |  | GLYMA19G40590 | 2.120105 |  |
| LPAT | Lysophosphatidyl acyltransferase | GLYMA11G12830 | 1.753234 | Up |
| PAP | Phosphatidic acid phosphatase | GLYMA09G18450 | 1.153165 | UP |
|  |  | GLYMA20G25650 | 1.557043 |  |
| DGAT | Diacylglycerol O-acyltransferase | GLYMA09G07520 | 1.611215 | Up |
|  |  | GLYMA01G36011 | 1.06689 | Down |
|  |  | GLYMA18G49230 | -2.15366 |  |
|  |  | GLYMA09G37430 | -1.41669 |  |
| PDAT | Phospholipid:diacylglycerol acyltransferase | GLYMA12G08915 | -1.85558 | Down |
|  |  | GLYMA17G05910 | 3.180158 | Up |
| TAGL | Triacylglycerol lipase | GLYMA12G05840 | -3.56353241 | Down |
|  |  | GLYMA10G16530 | -1.41669102 |  |
|  |  | GLYMA16G06230 | 1.975626405 | Up |
| DAGL | Diacylglycerol lipase | GLYMA09G34250 | -1.71713134 | Down |
| MAGL | Monoacylglycerol lipase | GLYMA08G19060 | 1.8687112 | Up |
| PLA | Phospholipase A1,2 | GLYMA11G03970 | 4.6043706 | Up |
|  |  | GLYMA05G05230 | 3.37772485 |  |
|  |  | GLYMA08G10600 | 3.22716517 |  |
|  |  | GLYMA01G41450 | 3.0386362 |  |
|  |  | GLYMA08G03260 | 2.97562641 |  |
|  |  | GLYMA03G31570 | 1.97562641 |  |
|  |  | GLYMA13G30430 | 1.65369831 |  |
| PLC | Phospholipase C | GLYMA11G35310 | 3.12246779 | Up |
|  |  | GLYMA14G37290 | 1.49019958 |  |
|  |  | GLYMA02G42410 | 1.16827148 |  |
| PLD | Phospholipsae D | GLYMA15G02710 | 1.75323398 | Up |
|  |  | GLYMA07G08740 | 1.34633885 |  |
| FAD 7 | Fatty acid desaturase 7 | GLYMA07G18350 | 5.457368 | Up |
|  |  | GLYMA18G43210 | 2.195388 |  |
| FAD8 | Fatty acid desaturase 8 | GLYMA01G29630 | 1.238661 | Up |
| FAD3 | Fatty acid desaturase 3 | GLYMA14G37350 | -1.25095 | Down |
|  |  | GLYMA02G39230 | -1.12718 |  |
| OLE1 | OLEOSIN1 | GLYMA19G13060 | 1.430178 | Up |
|  |  | GLYMA05G07880 | 1.685567 |  |

**Supplementary Table S3 . List of genes encoding enzymes of TCA cycle**

| **Glyma ID** | **GUS** | **GmLEC2** | **FC** | **Log_2_FC** | **Gene description** |
| --- | --- | --- | --- | --- | --- |
| GLYMA20G23200 | 1.886 | 6.360 | 3.37 | 1.75 | 2-Oxoglutarate dehydrogenase A |
| GLYMA08G03840 | 0.943 | 6.360 | 6.74 | 2.75 | Malate dehydrogenase A |
| GLYMA06G09220 | 57.544 | 556.527 | 9.67 | 3.27 | Malate dehydrogenase B |
| GLYMA12G33820 | 340.549 | 803.519 | 2.35 | 1.23 | Pyruvate carboxylase A |
| GLYMA01G22840 | 0.943 | 2.120 | 2.24 | 1.16 | Pyruvate carboxylase B |
| GLYMA13G34560 | 82.071 | 165.368 | 2.01 | 1.01 | Pyruvate carboxylase C |
| GLYMA03G41190 | 373.566 | 138.866 | 0.371 | -1.42 | Pyruvate carboxylase kinase |
| GLYMA08G17010 | 21.697 | 97.524 | 4.49 | 2.16 | Citrate synthase |
| GLYMA14G36540 | 1769.724 | 813.060 | 0.45 | -1.12 | Pyruvate dehydrogenase –mitochondrila |
| GLYMA14G39160 | 6523.262 | 2935.285 | 0.44 | -1.15 | Isocitrate dehydrogenase |

**Sequence data:**

>GmLEC2a

ATGGAAAACTTTTTTGTGCCATTTTTAAAAAAAAACCCCAACCCATCAATCACCACTACTGGTGGCAATGGCTCATCTTCATCAAACCAAACAATCCTTGTACAACCAAGCACGTATCCTCAAAATTTCCCTTACAATACTAGTGTAAAACTTAACTTTCCAGAACAACCTTATTTCATTCCTTTGTATCCCTTTCCAACAGGACAAGTTAGCTTTTCCAATCAACCCTATGGAATGCCAAATTCGGAACTTCAAGGTTCGAGGGCATGCATGACCAAAGCTACAAGGGAGAGATGGAGACAAGTAAGACAAAGGAGTAAAAATTCTACTCTTGTCGCTCCTAATTCAGTTCTAGAAAGGACAACAAGAGAACAATTTGTTCCTAATGGAGGGTCAAATGTGAGGATCACAGTCAAACAACACAATGCAACCAAGTTTTTTAACACCCCAAACGGGAAGAAGCTAGAAGAAATTTTGACAAAGAAGTTGAATAATAGTGATGTTGGCGTCCTAGGCCGCATTGTGCTCCCAAAGAGAGGGGCTGAGGATAAGCTTCCGACACTGTGGAAGAAGGAAGGAATCAATATTGTACTAAAGGATGTATATTCTGAGATTGAATGGAGCATCAAATACAAGTACTGGACTAATAACAAAAGCAGAATGTATATTCTTGATAATATAGGGGATTTTGTTAACCATTATAAACTTCAAGCAGGAGATTTCATAACCCTTTGCAAGGACGAGTTGAAAAATCTGTATGTGTCGGCTAGAAAGGATCACGAAAATCTAGAAGAATCTAAGTCCTCGTCAAACACAGGAATGTCACATGAACCAGATGCATATTTAGCTTACTTGACGAAGGAACTTGGCCATAAGGGGAAAGCAGAAGCTGCCAACAACCTTTTGAACAATGTTGAGGAAGAGGCACCATATCAAGCAAATCAATTACATCAATTTATGCCGATGAACAATATTGTGGGGGAGGGGGCATCAAACCAAGCAATTCAAGAAGCCGCACCAGCAGCACCCGTCAATGTTGATCAAGAAAACAAAGTTGTTGACGACGATGATGATGATATCTATGGTGGCCTTGACAATACTTTCGAAATTGGAAATACTTATCAAATTTGGTAG

>Glyma.20G035800.1 CDS
ATGGAAAACTTTTTTGTGCCATTTTTAAAAAAAAACCCCAACCCATCAATCACCACTACTGGTGGCAATGGCTCATCTTCATCAAACCAAACAAGCCTTG
TACAACCAAGCACATATCCTCAAAATTTCCCTTACAATACTAGTGTAAAACTTAACTTTCCAGAACAACCTTATTTCATTCCTTTGTATCCCTTTCCAAC
AGGACAAGTTAGCTTTTCTAATCAACCCTATGGAATGCCAAATTCGGAACTTCAAGGTTCGAGGGCATGCATGACCAAAGCTACAAGGGAGAGATGGAGA
CAAGTAAGACAAAGGAGTAAAAATTCTACTCTTGTCGCTCCTAATTCAGTTCTAGAAAGGACAACAAGAGAACAATTTGTTCCTAATGGAGGGTCAAATG
TGAGGATCACAGTCAAACAACACAATGCAACCAAGTTTTTTAACACCCCAAACGGGAAGAAGCTAGAAGAAATTTTGACAAAGAAGTTGAATAATAGTGA
TGTTGGCGTCCTAGGCCGCATTGTGCTCCCAAAGAGAGAGGCTGAGGATAAGCTTCCGACACTGTGGAAGAAGGAAGGAATCAATATTGTACTAAAGGAT
GTATATTCTGAGATTGAATGGAGCATCAAATACAAGTACTGGACTAATAACAAAAGCAGAATGTATATTCTTGATAATACAGGGGATTTTGTTAACCATT
ATAAACTTCAAACAGGAGATTTCATAACCCTTTACAAGGACGAGTTGAAAAATCTGTATGTGTCGGCTCGAAAGGATCAAGAAAATCTAGAAGAATCTAA
GTCCTCGTCAAACACAGGAATGTCACATGAACCAGATGCATATTTAGCTTACTTGACGAAGGAACTTAGCCATAAGGGGAAAGCAGAAGCTGCCAACAAC
CTTTTGAACAATGTTGAGGAAGAGGCACCAAATCAAGCAAATCAATTACATCAATTCATGCCGATGAACAATATTGTTGGGGAGGGGGCATCAAACCAAG
CAATTCAAGAAGCCGCACCAGCCGCACCCGTCAATGTTAATCAAGAAAACAAAGTTGTTGACGACGATGATGATGATATCTATGGTGGCCTTGACAATAT
TTTCGAAATTGGAAATACTTATCAAATTTGGTAG

>Glyma.20G035700.1 CDS
ATGGAAAACTTTTTTGTGCCATTTTTTAAAAAAAACCCCAACCCATCAATCACCACTACTGGTGGCAGTGGCTCATCTTCATCAAACCAAACAAGCCTTG
TACAACCAAGCACATATCCTCAAAATTTCCCTTACAATACTAGTGAAAAACTTAACTTTCCAGAACAACCTTATTTCATTCCTTTGTATCCCTTTCCAAC
AGGACAAGTTAGCTTTTCTAATCAACCCTATGGAATGCCAAATTCGGAACTTCAAGGTTCGAGGGCATGCATGACCAAAGCTACAAGGGAGAGATGGAGA
CAAGTAAGACAAAGGAGTAAAAATTCTACTCCTGTCGCTCCTAATTCAATTCTAGAAGGGACAACAAGGGAACAATTTGTTCCTAATGGAGGGTCAAATG
TGAGGATCACAGTCAAACAACACAATGCAACCAAGTTTTTTAACACCCCAAACGGGAAGAAGCTAGAAGAAATTTTGACAAAGAAGTTGAATAAGAGTGA
TGTTGGCGTCCTAGGCCGCATTGTGCTCCCAAAGAGAGAGGCTGAGGATAAGCTTCCGACACTGTGGAAGAAGGAAGGAATCAATATTGTACTAAAGGAT
GTATATTCTGAGATTGAATGGAGCATCAAATACAAGTACTGGACTAATAACAAAAGCAGAATGTATATTCTTGATAATATAGGGGATTTTGTTAACCATT
ATAAACTTCAAGCAGGAGATTTCATAACCCTTTACAAGGACGAGTTGAAAAATCTGTATGTGTCGGCTAGAAAGGATCACGAAAATCTAGAAGAATCTAA
GTCCTCGTCAAACACAGGAATGTCACATGAACCAGATGCATATTTAGCTTACTTGACGAAGGAACTTGGCCATAAGGGGAAAGCAGAAGCTGCCAACAAC
CTTTTGAACAATGTTGAGGAAGAGGCACCATATCAAGCAAATCAATTACATCAATTCATGCCGATGAACAATATTGTGGGGGAGGGGGCATCAAACCAAG
CAATTCAAGAAGCCGCACCAGCAGCACCCGTCAATGTTGATCAAGAAAACAAAGTTGTTGACGACGATGATGATGATATCTATGGTGGCCTTGACAATAT
TTTCGAAATTGGAAATACTTATCAAATTTGGTAG

> GmLEC2a

MENFFVPFLKKNPNPSITTTGGNGSSSSNQTILVQPSTYPQNFPYNTSVKLNFPEQPYFI

PLYPFPTGQVSFSNQPYGMPNSELQGSRACMTKATRERWRQVRQRSKNSTLVAPNSVLER

TTREQFVPNGGSNVRITVKQHNATKFFNTPNGKKLEEILTKKLNNSDVGVLGRIVLPKRG

AEDKLPTLWKKEGINIVLKDVYSEIEWSIKYKYWTNNKSRMYILDNIGDFVNHYKLQAGD

FITLCKDELKNLYVSARKDHENLEESKSSSNTGMSHEPDAYLAYLTKELGHKGKAEAANN

LLNNVEEEAPYQANQLHQFMPMNNIVGEGASNQAIQEAAPAAPVNVDQENKVVDDDDDDI

YGGLDNTFEIGNTYQIW

>Glyma.20G035800.1
MENFFVPFLKKNPNPSITTTGGNGSSSSNQTSLVQPSTYPQNFPYNTSVKLNFPEQPYFIPLYPFPTGQVSFSNQPYGMPNSE
LQGSRACMTKATRERWRQVRQRSKNSTLVAPNSVLERTTREQFVPNGGSNVRITVKQHNATKFFNTPNGKKLEEILTKKLNNSDVGVLGRIVLPKREAED
KLPTLWKKEGINIVLKDVYSEIEWSIKYKYWTNNKSRMYILDNTGDFVNHYKLQTGDFITLYKDELKNLYVSARKDQENLEESKSSSNTGMSHEPDAYLA
YLTKELSHKGKAEAANNLLNNVEEEAPNQANQLHQFMPMNNIVGEGASNQAIQEAAPAAPVNVNQENKVVDDDDDDIYGGLDNIFEIGNTYQIW*

>Glyma.20G035700.1
MENFFVPFFKKNPNPSITTTGGSGSSSSNQTSLVQPSTYPQNFPYNTSEKLNFPEQPYFIPLYPFPTGQVSFSNQPYGMPNSE
LQGSRACMTKATRERWRQVRQRSKNSTPVAPNSILEGTTREQFVPNGGSNVRITVKQHNATKFFNTPNGKKLEEILTKKLNKSDVGVLGRIVLPKREAED
KLPTLWKKEGINIVLKDVYSEIEWSIKYKYWTNNKSRMYILDNIGDFVNHYKLQAGDFITLYKDELKNLYVSARKDHENLEESKSSSNTGMSHEPDAYLA
YLTKELGHKGKAEAANNLLNNVEEEAPYQANQLHQFMPMNNIVGEGASNQAIQEAAPAAPVNVDQENKVVDDDDDDIYGGLDNIFEIGNTYQIW*
